# Anillin regulates breast cancer cell migration, growth, and metastasis by non-canonical mechanisms involving control of cell stemness and differentiation

**DOI:** 10.1186/s13058-019-1241-x

**Published:** 2020-01-07

**Authors:** Dongdong Wang, Nayden G. Naydenov, Mikhail G. Dozmorov, Jennifer E. Koblinski, Andrei I. Ivanov

**Affiliations:** 1 Department of Human and Molecular Genetics, Massey Cancer Center, Virginia Commonwealth University, Richmond, VA 23298 USA; 2 Present address: Department of Pathology and Translational Pathobiology, Louisiana State University Health Sciences Center, Shreveport, LA 71103 USA; 3 Department of Inflammation and Immunity, Lerner Research Institute of Cleveland Clinic Foundation, 9500 Euclid Avenue, NC22, Cleveland, OH 44195 USA; 4 Department of Biostatistics, Massey Cancer Center, Virginia Commonwealth University, Richmond, VA 23298 USA; 5 Department of Pathology, Massey Cancer Center, Virginia Commonwealth University, Richmond, VA 23298 USA

**Keywords:** Actin cytoskeleton, Invasion, E-cadherin, Actin-binding proteins, Keratins, Stem cells

## Abstract

**Background:**

Breast cancer metastasis is driven by a profound remodeling of the cytoskeleton that enables efficient cell migration and invasion. Anillin is a unique scaffolding protein regulating major cytoskeletal structures, such as actin filaments, microtubules, and septin polymers. It is markedly overexpressed in breast cancer, and high anillin expression is associated with poor prognosis. The aim of this study was to investigate the role of anillin in breast cancer cell migration, growth, and metastasis.

**Methods:**

CRISPR/Cas9 technology was used to deplete anillin in highly metastatic MDA-MB-231 and BT549 cells and to overexpress it in poorly invasive MCF10AneoT cells. The effects of anillin depletion and overexpression on breast cancer cell motility in vitro were examined by wound healing and Matrigel invasion assays. Assembly of the actin cytoskeleton and matrix adhesion were evaluated by immunofluorescence labeling and confocal microscopy. In vitro tumor development was monitored by soft agar growth assays, whereas cancer stem cells were examined using a mammosphere formation assay and flow cytometry. The effects of anillin knockout on tumor growth and metastasis in vivo were determined by injecting control and anillin-depleted breast cancer cells into NSG mice.

**Results:**

Loss-of-function and gain-of-function studies demonstrated that anillin is necessary and sufficient to accelerate migration, invasion, and anchorage-independent growth of breast cancer cells in vitro. Furthermore, loss of anillin markedly attenuated primary tumor growth and metastasis of breast cancer in vivo. In breast cancer cells, anillin was localized in the nucleus; however, knockout of this protein affected the cytoplasmic/cortical events, e.g., the organization of actin cytoskeleton and cell-matrix adhesions. Furthermore, we observed a global transcriptional reprogramming of anillin-depleted breast cancer cells that resulted in suppression of their stemness and induction of the mesenchymal to epithelial trans-differentiation. Such trans-differentiation was manifested by the upregulation of basal keratins along with the increased expression of E-cadherin and P-cadherin. Knockdown of E-cadherin restored the impaired migration and invasion of anillin-deficient breast cancer cells.

**Conclusion:**

Our study demonstrates that anillin plays essential roles in promoting breast cancer growth and metastatic dissemination in vitro and in vivo and unravels novel functions of anillin in regulating breast cancer stemness and differentiation.

## Background

Breast cancer is the most frequently diagnosed cancer in women worldwide and is highly lethal when it progresses to the metastatic stage. The 5-year survival rate for breast cancer patients presenting with a locally contained primary tumor is 99%, but is only 27% for those presenting with metastatic disease [1]. Accelerated cancer cell motility that allows them to invade the surrounding tissues and disseminate throughout the body is a hallmark of cancer metastasis [2–4]. Remodeling of the intracellular cytoskeleton represents the most important event in cell migration because the cytoskeleton creates the infrastructure and provides the driving forces in motile cells [4–6]. All major cytoskeletal elements, such as actin filaments, microtubules, and septin filaments, are essential for cell migration [4, 7]. How these cytoskeletal elements cooperate to drive breast cancer cell invasion and metastasis remains poorly understood. Importantly, current anticancer drugs that interfere with the cytoskeleton narrowly target microtubules, and this approach suffers from severe side effects and the development of drug resistance. No therapeutic approaches exist to interfere with the actin cytoskeleton or the septin cytoskeleton, which are essential for breast cancer growth and metastasis [4, 8–10]. However, both actin filaments and septin filaments have important functions in normal cells, and they are likely to be poor targets for anticancer drug development. A more feasible approach would be to identify and inhibit common upstream regulators of major types of the cytoskeleton that are hyperactivated in invasive breast cancer cells.

Anillin is a unique scaffolding protein that interacts with and regulates different cytoskeletal elements during specific stages of the cell cycle [11, 12]. Anillin directly binds to actin filaments and induces filament bundling [13, 14]. It also interacts with a key actin motor, non-muscle myosin (NM) II, and controls the localization and activity of NM II in mitotic cells [15–17]. In addition to direct binding, anillin also regulates the actomyosin cytoskeleton indirectly, by modulating intracellular signaling cascades. Specifically, anillin is known to either inhibit or activate members of the Rho family of small GTPases, such as RhoA, RhoG, and Rac1 [16, 18–20]. Anillin-dependent regulation of the cytoskeleton is not limited to actomyosin filaments since this scaffolding protein regulates the assembly and dynamics of septin filaments and microtubules [15, 21, 22]. Anillin plays a prominent role in cytokinesis by controlling cleavage furrow positioning and ingression during the separation of a dividing cell into daughter cells [11, 12]. Moreover, recent studies reveal unanticipated anillin functions in non-dividing cells, which include regulation of cell-cell and cell-matrix adhesions [18, 19, 23–25].

A number of clinical studies demonstrate anillin overexpression in human cancers, predominantly in breast, pancreatic, colon, lung, gastric, and liver carcinoma [19, 26–32]. In breast cancer, anillin was found to be markedly upregulated at both mRNA and protein levels [26, 28, 31, 33] and was identified among the limited number of proteins upregulated during transition from ductal carcinoma in situ to invasive ductal carcinoma [34]. High anillin expression was associated with more aggressive estrogen and progesterone receptor-deficient (ER-, PR-) breast tumors but appeared to be independent of the human epidermal growth factor receptor 2 (HER2) status [28, 31, 35]. Furthermore, a meta-analysis of gene and protein expression data demonstrated that high anillin expression is a prognostic marker of poor survival in breast cancer patients [28, 31]. Although it has been shown that anillin depletion attenuates breast cancer cell motility and proliferation in vitro [28, 33], it is unknown if anillin overexpression alone is capable of promoting migration, invasion, and growth of breast cancer cells. More importantly, none of the previous studies addressed the involvement of anillin in breast cancer development and metastasis in vivo. Finally, molecular mechanisms underlying anillin functions in cancer cells have not been elucidated. The present study was designed to fill these important gaps in knowledge by investigating the roles and mechanisms of anillin-driven breast carcinogenesis. We report that anillin promotes breast cancer motility and growth in vitro and in vivo by non-canonical mechanisms involving regulation of cancer cell stemness and differentiation.

## Methods

### Antibodies and other reagents

The following primary monoclonal (mAb) and polyclonal (pAb) antibodies were used to detect cytoskeletal, membrane, and signaling proteins: anti-anillin (A301-405A and A301-406A) pAbs (Bethyl Laboratories, Montgomery, TX); Keratin 14 mAb and Keratin 17 pAb (Proteintech, Rosemont, IL), anti-E-cadherin, calponin-1, L-caldesmon, and vimentin mAbs (BD Biosciences, San Jose, CA); anti-P-cadherin mAb MAB 861 and E-cadherin pAb (R & D Systems, Minneapolis, MN); anti-cadherin 11 and GAPDH pAbs, anti N-cadherin, cleaved caspase 3, and Ki-67 mAbs (Cell Signaling Technology, Danvers, MA); anti-SM22-pAb (Abcam, Cambridge, MA). PE-conjugated anti-CD4 and APC-conjugated anti-CD24 mAbs were obtained from BioLegend (San Diego, CA). Alexa Fluor-488-conjugated donkey anti-rabbit and Alexa Fluor-555-conjugated donkey anti-mouse secondary antibodies and Alexa Fluor-488 and 555-labeled phalloidin were obtained from Thermo Fisher. Horseradish peroxidase-conjugated goat anti-rabbit and anti-mouse secondary antibodies were acquired from Bio-Rad Laboratories. pFULT-tdTomato-Luciferase virus particles were bought from the DNA/RNA Delivery Core facility of the Northwestern University School of Medicine. All other chemicals were of the highest purity and obtained from Millipore-Sigma or Thermo Fisher.

### Cell culture

MDA-MB-231, MDA-MB-468, MCF7, BT474, Hs578T, BT549, and HEK293 cells were acquired from the American Type Culture Collection (Manassas, VA). MCF10A, MCF10AneoT, and MCF10A1d.cl1 cells were purchased from the Barbara Ann Karmanos Cancer Institute, Wayne State University (Detroit, MI). MDA-MB-231, MDA-MB-496, MCF7, BT474, Hs578T, and HEK293 cells were grown in Dulbecco’s modified Eagle’s medium (DMEM) supplemented with 10% fetal bovine serum and penicillin/streptomycin. BT549, cells were grown in RPMI, supplemented with 10% fetal bovine serum, insulin, and penicillin/streptomycin. MCF10A cells were grown in DMEM/F12 containing 5% horse serum, human EGF, human insulin, hydrocortisone, penicillin/streptomycin, and cholera toxin. All cells were maintained at 37 °C in an atmosphere containing 5% CO_2_. The cells were grown in T75 flasks and were seeded on collagen-coated coverslips or six-well plastic plates for immunolabeling and biochemical experiments, respectively.

### CRISPR/Cas9-mediated gene editing for anillin knockout and overexpression

A stable knockout of anillin in MDA-MB-231 and BT549 cells was carried out using CRISPR-Cas9 technology. Different anillin-targeting single-guide RNAs (sgRNAs) or non-targeting sgRNAs were selected from two genome-wide sgRNA libraries generated by Zhang and Lander laboratories [36]. The selected sgRNA oligonucleotides (sequences are included in Additional file 1: Table S1A) were annealed and cloned into the BsmBI site of a lentiCRISPR v2 vector (Addgene, 52961) and the obtained constructs were verified by sequencing. Transfer plasmids possessing the annealed guide oligonucleotides were transformed into recombination-deficient One Shot™ Stbl3™ Chemically Competent *E. coli* (Thermo Fisher), and the amplified plasmids were isolated using a Qiagen midi prep plasmid isolation kit. Lentiviruses were produced by transfecting HEK-293T cells with a transfer lentiCRISPR v2 plasmid and packaging pLTR-G (Addgene, 17532) and pCD/NL-BH^*^DDD (Addgene, 17531) plasmids. Viral supernatants were collected 48 and 72 h after transfection and used to infect MDA-MB-231 and BT549 cells in the presence of polybrene. After 48 h of the infection, the lentivirus-containing medium was replaced with fresh cell culture medium containing puromycin (5 μg/ml for MDA-MB-231 and 1 μg/ml for BT549 cells) and puromycin-resistant cells were collected after 7-day selection.

For the anillin overexpression in MCF10AneoT cells, a CRISPR/Cas9-based transcriptional gene activation system [37] obtained from Santa Cruz Biotechnology (sc-403342-LAC) was used. Cells were transfected with either anillin-activating lentiviral particles or control lentiviral particles (sc-108084) in the presence of polybrene, according to the manufacturer’s protocol. After 48 h of viral transduction, stable cell lines were selected by co-treatment with three different antibiotics: blasticidin (10 μg/mL), puromycin (5 μg/mL), and hygromycin B (300 μg/mL).

As an alternative approach, anillin was overexpressed in MCF10AneoT cells using a pTK88-GFP-Anillin retroviral plasmid (Addgene, 46354) with a pWZL-GFP plasmid (Addgene, 12269) as a control. Retrovirus packaging was performed by transfecting those plasmids together with packaging plasmids, ENV and GagPol, into 60% confluent Phoenix cells using a TransIT2020 transfection reagent. Retroviruses were collected at 48 and 72 h post transfection. MCF10AneoT cells plated at 30% confluency were infected by retroviruses with 5 μg/mL polybrene. After 72 h of viral transduction, stable GFP-anillin expressed cell lines were selected by flow cytometry.

### RNA interference

siRNA-mediated knockdown of either E-cadherin or P-cadherin in control and anillin-depleted breast cancer cells was carried as previously described [38, 39]. E-cadherin was depleted by using Dharmacon siRNA duplexes (duplex 1, D003877-02; duplex 2, D003877-05), whereas P-cadherin was depleted using specific Dharmacon siRNA SmartPool (L003823-00). A non-targeting siRNA duplex 2 was used as a control. Cells were transfected using DharmaFECT 1 reagent in Opti-MEM I medium (Thermo Fisher) according to the manufacturer’s protocol, with a final siRNA concentration of 50 nM. Cells were used in the experiments on days 3 and 4 post transfection.

### Scratch wound assay

Confluent breast cancer cell monolayers were mechanically wounded by making a thin scratch with a 200-μl pipette tip. The bottom of the well was marked in a cell-free area to define the position of the wound. Images at the marked region were acquired at the indicated times after wounding using an inverted bright-field microscope equipped with a camera. The percentage of wound closure was calculated using a TScratch software [40].

### Matrigel invasion assay

A Matrigel invasion assay was performed using BD Biocoat invasion chambers (BD Biosciences). Cells were disassociated from the culture dish using a TrypLE Express reagent (Thermo Fisher), counted, resuspended into a serum-free medium, and added to the upper chamber at a concentration of 5 × 10^4^ cells per chamber. Complete cell culture medium containing 10% FBS as a chemoattractant was added to the lower chamber, and cells were allowed to invade through Matrigel for 24 h at 37 °C. The Matrigel plugs were washed with phosphate-buffered saline (PBS) and fixed with methanol, and non-migrated cells were removed from the top of the gel using cotton swabs. The invaded cells were stained with DAPI, visualized by a fluorescence microscope, and counted by using an ImageJ program (National Institute of Health, Bethesda, MD).

### Extracellular matrix adhesion assay

Cell-matrix adhesion assay was performed as previously described [41]. Briefly, control, anillin-depleted, and anillin-overexpressing cells were dissociated by the TrypLE Express reagent, counted with a hemocytometer, and resuspended in the complete medium. 3 × 10^4^ cells were seeded to each well of 24-well plates coated with either collagen I, fibronectin, collagen IV, or laminin and were allowed to adhere for 30 min at 37 °C. After incubation, unattached cells were removed and attached cells were fixed and stained with a DIFF stain kit (IMEB Inc., San Marcos, CA). Images of adherent cells were captured using the bright-field microscope, and the number of adhered cells was determined using the ImageJ software.

### Cell proliferation and soft agar colony formation assays

To examine anchorage-dependent cell proliferation, MDA-MB-231 and MCF10AneoT cells were seeded on 60-mm cell culture dishes at the density of 4 × 10^4^ and 1 × 10^5^ cells per dish, respectively. Cells were allowed to proliferate for the indicated times and stained with 0.4% Trypan Blue Solution, and the number of live cells was counted using a hemocytometer.

The anchorage-independent cell growth was examined by using a soft agar colony formation assay according to a published protocol [42]. Briefly, a 2× cell culture medium was mixed with an equal volume of 1.2% noble agar (BD Biosciences) to form a solid 0.6% agar layer at the bottom of a six-well plate. Cells were resuspended in the 2× culture medium and mixed with an equal volume of 0.8% agar to obtain an upper 0.4% agar layer containing 1 × 10^4^ cells per well. These agar plates were cultured at 37 °C with the addition of fresh medium every other day. After 40 days of culturing, these agar plates were washed with PBS and stained with methylene blue (0.06 g/mL) dissolved in 1.5% (v/v) buffered glutaraldehyde. Cell colonies were imaged and counted using ImageJ software.

### Mammosphere formation assay

A Mammosphere formation assay was performed using a MammoCult™ Human Medium Kit (STEMCELL Technologies, Cambridge, MA) according to the kit manual. Briefly, cells were grown to 80–90% confluency, rinsed with PBS, and scraped from the dish by using a sterile cell scraper. Harvested cells were transferred into the MammoCult medium and centrifuged, and the cell pellet was resuspended in the MamoCult medium. Cells (1 × 10^4^) were plated in each well of a six-well ultra-low adherent plate (STEMCELL Technologies) and incubated at 37 °C for 7 days. Mammospheres were imaged with a bright-field microscope. ImageJ software was used to quantify the number and the size of spheres larger than 60 μm in diameter.

### Flow cytometry analysis

Control, anillin-depleted, and overexpressing cells were lifted from plates using the TrypLE Express reagent and dual-labeled with a PE-conjugated anti-CD44 antibody and an APC-conjugated anti-CD24 antibody. Labeled cells were analyzed in the Lerner Research Institute Flow Cytometry Core using a BD LSRII flow cytometer and FACSDiva (Becton-Dickinson) software. APC fluorescence was excited by the red laser (639 nm) and detected with the 660/20 filter, whereas PE fluorescence was excited by the green laser (532 nm) and detected with the 575/26 filter.

### Immunoblotting analysis

Cells were homogenized in RIPA lysis buffer (20 mM Tris, 50 mM NaCl, 2 mM EDTA, 2 mM EGTA, 1% sodium deoxycholate, 1% Triton X-100 (TX-100), and 0.1% SDS, pH 7.4), containing a protease inhibitor cocktail (1:100, Sigma) and phosphatase inhibitor cocktails 1 and 3 (both at 1:200, Sigma). Lysates were cleared by centrifugation (20 min at 14,000×*g*), diluted with 2× SDS sample buffer and boiled. SDS-PAGE and immunoblotting were conducted by standard protocols with an equal amount of total protein (10 or 20 μg) per lane. Results shown are representative immunoblots of at least three independent experiments. Protein expression was quantified by densitometry, and signal intensities were calculated using ImageJ software. The densitometry data are presented as normalized values, with the expression level of control groups set at 1 arbitrary unit.

### Immunofluorescence labeling and confocal microscopy

MDA-MB-231 cell monolayers plated on collagen-coated coverslips were fixed with 4% paraformaldehyde and permeabilized with 0.5% Triton X-100 at room temperature. Fixed cells were blocked for 60 min in PBS containing 1% bovine serum albumin. Cells were incubated with the appropriate concentrations of primary antibodies in blocking solution for 60 min, washed with blocking buffer, incubated with Alexa dye-conjugated secondary antibodies and Alexa-labeled phalloidin (to detect filamentous (F) actin) for 60 min, washed, and mounted on slides with a ProLong Antifade mounting reagent (Thermo Fisher). Labeled cell monolayers were observed using a Zeiss LSM 700 Laser Scanning Confocal Microscope (Carl Zeiss Microimaging Inc.; Thornwood; NY). The Alexa Fluor 488 and 555 signals were imaged sequentially in frame-interlace mode to eliminate crosstalk between channels. Image analysis was conducted using imaging software ZEN 2011 (Carl Zeiss Microscopy Inc.) and Adobe Photoshop. Images shown are representative of at least three experiments. Multiple images were captured from each slide.

### Rho GTPase activation assays

RhoA activity in control and anillin-depleted MDA-MB-231 cells was determined by the enzyme-linked immunosorbent assay using a G-LISA activation assay kit (Cytoskeleton Inc., Denver, CO). The assay was performed according to the manufacturer’s protocol, with the internal active RhoA standard used as a positive control. The sample absorbance at 490 nm was measured using a microplate reader. Rac1 activity was examined using a pull-down assays kit from Cell Biolabs (San Diego, CA) according to the manufacturer’s instructions. The amount of precipitated Rac1, as well as total Rac1 in cell lysates, was determined by immunoblotting analysis.

### RNA sequencing analysis

Total RNA was extracted from either control or anillin-deficient (two different CRISPR probes, referred hereafter as CRISPR1 and CRISPR2) MDA-MB-231 cell lines using mirVana miRNA Isolation Kit (Thermo Fisher Scientific, Cat #AM1560). The RNA quality was determined by Bioanalyzer (Agilent, Santa Clara, CA). To isolate the polyA RNA, NEBNext Poly(A) mRNA Magnetic Isolation Module (New England BioLabs, Ipswich, MA) combined with SMARTer Apollo NGS library preparation system (Takara, Mountain View, CA) was used with a total of 1 μg of good-quality total RNA as input. The NEBNext Ultra Directional RNA Library Prep Kit (New England BioLabs) was then used for polyA RNA library preparation, which is a dUTP-based stranded library. The library was indexed and amplified under the PCR cycle number of 11. After library Bioanalyzer QC analysis and quantification, individually indexed and compatible libraries were proportionally pooled and sequenced using an Illumina HiSeq 1000 sequencer. Under the sequencing setting of single read 1 × 51 bp, about 25 million pass filter reads per sample were generated.

Two biological replicates for each sample were sequenced. One set of replicates had an insufficient number of reads and was additionally sequenced. These additional sequences were concatenated with the under-sequenced samples.

Quality control at each processing step was performed using the FastQC tool v.0.11.2 (quality base calls, CG content distribution, duplicate levels, complexity level) https://www.bioinformatics.babraham.ac.uk/projects/fastqc/. Single-end reads were adapter-trimmed using Trimmomatic v.0.33 [43] and aligned using the subread v.1.6.2 aligner and the latest assembly of the human genome (GRCh38/hg38). Gene counts were obtained using the feature-Count v1.6.2 software and analyzed for differential expression using the edgeR v3.24.3 R package. The sequences from two replicates were merged together. Variability (batch effect) due to the “merged” status of one set of replicates was accounted for in the detection of differentially expressed genes. *P* values were corrected for multiple testing using a false discovery rate (FDR) multiple testing correction method [https://www.jstor.org/stable/2346101]. Genes at FDR < 0.1 were selected as differentially expressed. Gene set enrichment analysis [44] was performed on a list of 275 genes differentially expressed in both CRISPR experiments using the clusterProfiler v3.10.1 R package. All analyses were performed in the R/Bioconductor v3.5.3 statistical environment.

### Quantitative RT-PCR

Total RNA was isolated using the RNeasy mini kit (QIAGEN, Valencia CA), followed by DNase treatment to remove genomic DNA. Total RNA (1 μg) was reverse transcribed using the iScript cDNA synthesis kit (Bio-Rad Laboratories). Quantitative real-time PCR was performed using iTaq Universal SYBR Green Supermix (Bio-Rad Laboratories). Gene amplification was performed using a CFX96 Real-Time PCR System (Bio-Rad Laboratories) with the following reaction conditions: 1 cycle, 95 °C for 120 s; and 40 cycles, 95 °C for 1 s, and 60 °C for 20 s. A threshold cycle number for the gene of interest was calculated based on the amplification curve representing a plot of the fluorescence signal intensity versus the cycle number. The delta threshold cycle number was calculated as the difference between the threshold cycle number of the genes of interest between control and anillin knockout cells. Each value was normalized by the difference in the threshold cycle number for the housekeeping gene (GAPDH) amplification in the same samples. Primer sequences are included in Additional file 1: Table S1B.

### In vivo studies of tumor growth and metastasis

NOD-SCID-IL2 gamma-receptor null (NSG) mice were purchased from Jackson Laboratory (#005557), bred at Virginia Commonwealth University (VCU) Cancer Mouse Models Core Laboratory (generation F2), and housed under standard pathogen-free conditions with food and water available ad libitum. To monitor tumor growth and metastasis, control and anillin-depleted MDA-MB-231 cells were stably transduced with a pFULT-tdTomato-Luciferase expressing lentivirus. The cells were sorted by flow cytometry to obtain populations with similar tdTomato expression before injection. Similar level of luciferase was also determined before injection by a serial dilution of cells in 96-well plates, addition of luciferin, imaging with In Vivo Imaging System (IVIS) Spectrum, and analysis with Living Image software (PerkinElmer). To assess the effects of anillin on tumor growth and spontaneous metastases from the primary site, MDA-MB-231 cells (2.5 × 10^5^ cells/injection) were injected into the left and right sides of the lactiferous duct of the fourth mammary gland of the NSG mice (*n* = 12 animals per each experimental group) with the assistance of the VCU Cancer Mouse Models Core Laboratory. Calipers were used to measure tumor size, starting 1 week post-injection and continuing twice-weekly for the next 6 weeks. On day 42 after cell implantation, mice were subcutaneously injected with luciferin (150 mg/kg). Tumors, lungs, livers, kidneys, ovaries, and lymph nodes were dissected immediately after luciferin injection, and their luminescence intensity was measured using the IVIS Spectrum (PerkinElmer, Waltham, MA). Additionally, the weight of all dissected tumors was measured.

For the intracardiac injection, 2 × 10^5^ MDA-MB-231 cells were injected into the left ventricle of the NSG mouse heart (*n* = 16 mice per each experimental group). Bioluminescence images of the dorsal and ventral parts of the mouse body were taken weekly. Twenty-eight days after injection, mice were sacrificed, their lungs, livers, kidneys, ovaries, bones, and brains were dissected separately, and bioluminescence images were taken for each tissue. Image analysis and quantification were performed by a Living Image Software-IVIS Spectrum Series.

### Immunohistochemistry

Immunohistochemical (IHC) staining for cleaved caspase-3, E-Cadherin, and Ki-67 was performed in the Cancer Mouse Models Core Laboratory (CMMCL) with the Leica Bond RX autostainer using heat-induced epitope retrieval buffer 2 (Leica, EDTA pH 8.0). Stained slides were then imaged on the Vectra Polaris (Akova Biosciences) and quantified using InForm software (Akova Biosciences).

### Statistics

All numerical values from individual in vitro experiments were pooled and expressed as mean ± standard error of the mean (S.E) throughout. Obtained numbers were compared by two-tailed Student’s *t* test, with statistical significance assumed at *P* < 0.05. The numerical values from in vivo experiments were expressed as mean ± standard error of the mean (S.E) or scatter dot plot with a mean line. One-way ANOVA and Dunnett’s multiple comparisons test were performed for statistical significance assumed at *P* < 0.05.

## Results

### Modulation of anillin expression alters breast cancer cell migration, invasion, and soft agar growth in vitro

**Fig. 1:**
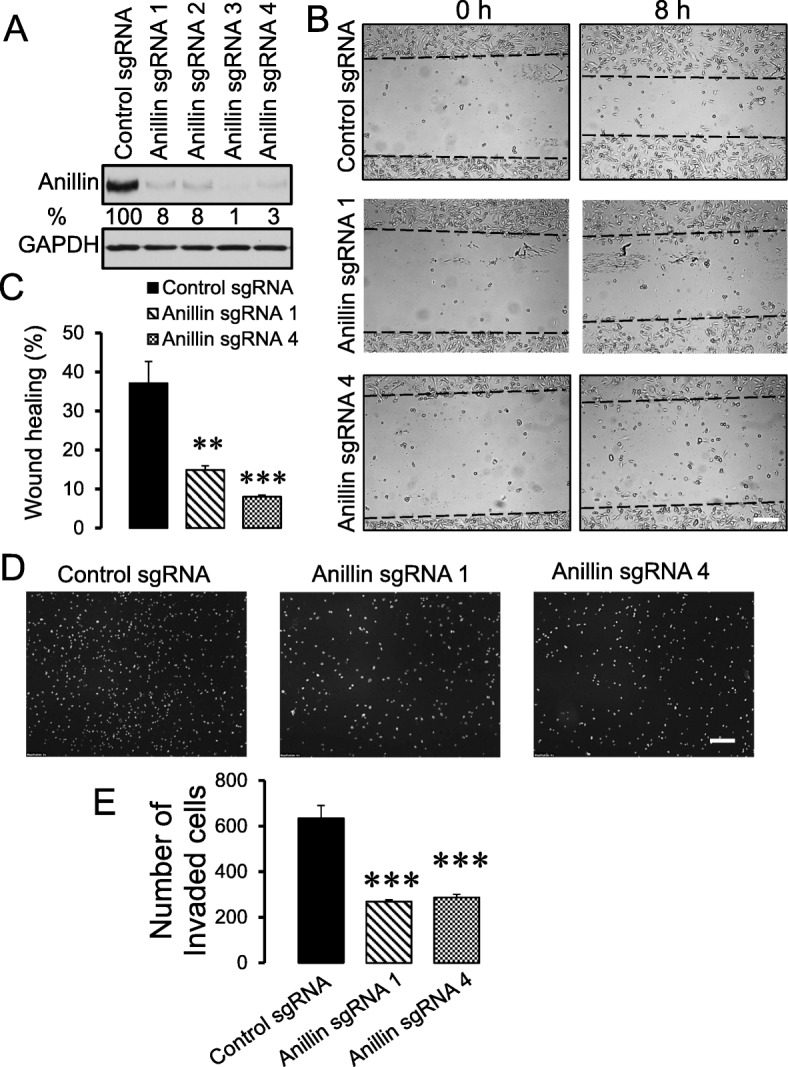
Loss of anillin expression inhibits collective migration and Matrigel invasion of breast cancer cells. Anillin expression was downregulated in MDA-MB-231 cells using CRISPR/Cas9-mediated gene editing. **a** Immunoblotting analysis shows the efficiency of anillin knockout by four different single-guide (sg) RNAs. **b**,**c** Representative images and quantification of the wound healing in control and anillin-depleted MDA-MB-231 cell monolayers at 8 h post-wounding. **d**,**e** Representative images and quantitative analysis of DAPI-labeled control and anillin-deficient MDA-MB-231 cells after 24 h invasion into Matrigel. Data are presented as mean ± SE (*n* = 3); **p < 0.01; ***p < 0.001, as compared to the control sgRNA-transfected group. Scale bars, 200 μm

**Fig. 2:**
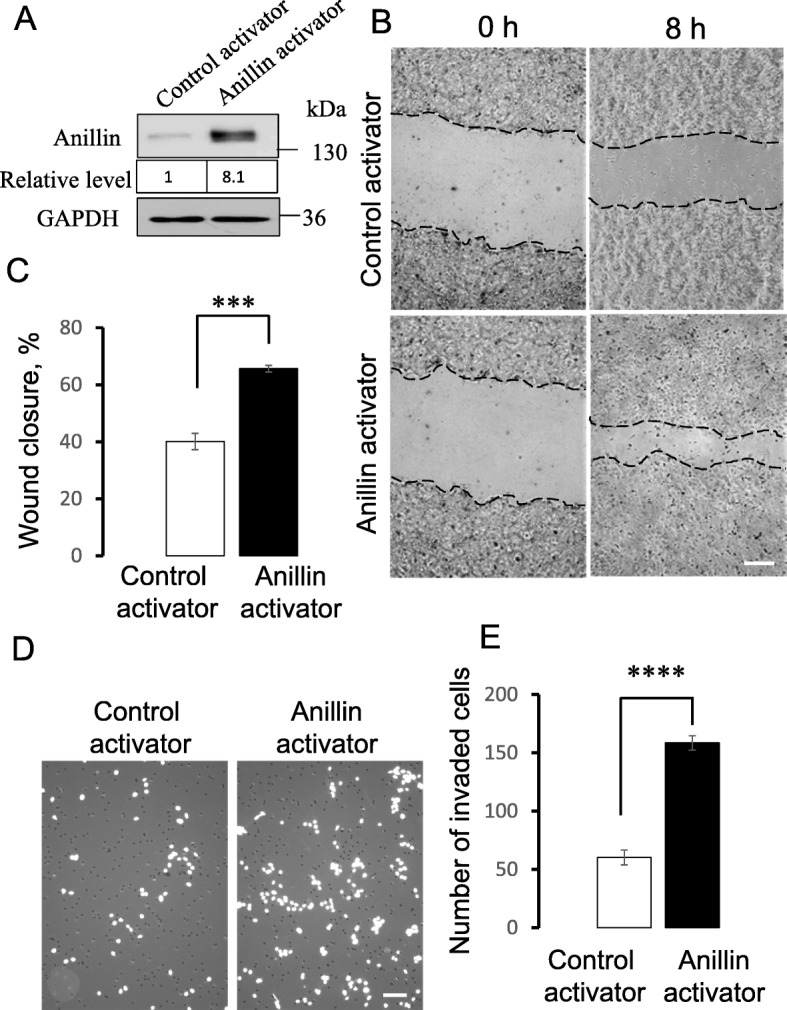
Increased anillin expression promotes collective migration and Matrigel invasion of breast cancer cells. Endogenous anillin expression was increased in MCF10AneoT cells using a specific CRISPR/Cas9-driving promoter-activation plasmid (anillin activator). **a** Immunoblotting analysis shows the magnitude of anillin overexpression. **b**,**c** Representative images and quantification of the collective migration of control and anillin-overexpressing MCF10AneoT cell monolayers at 8 h post-wounding. Scale bar, 100 μm. **d**,**e** Representative images and quantitative analysis of DAPI-labeled control and anillin-overexpressing cells after 24 h invasion into Matrigel. Data are presented as mean ± SE (*n* = 3); ***p < 0.001; ****p < 0.0001, as compared to the control group. Scale bar, 100 μm

**Fig. 3:**
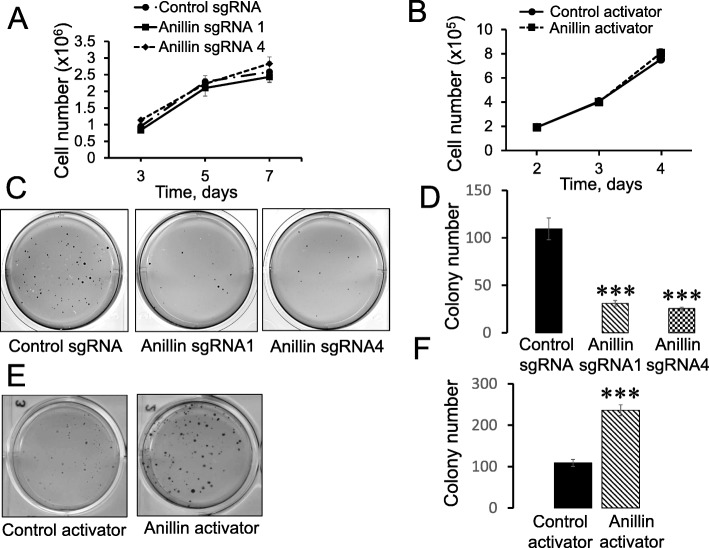
Anillin is not essential for anchorage-dependent cell proliferation, but is critical for the anchorage-independent growth of breast cancer cells. Control and anillin-depleted MDA-MB-231 cells (**a**), as well as control and anillin-overexpressing MCF10AneoT cells (**b**) were cultured on plastic plates. The number of cells at different time point was counted using a hemocytometer (*n* = 3). Control and anillin-depleted MDA-MB-231 cells (**c**,**d**), as well as control and anillin-overexpressing MCF10AneoT cells (**e**,**f**) were cultured on soft agar. The number of cell colonies was counted on days 40 (MDA-MB-231) or 35 (MCF10AneoT) after plating. Data is presented as mean ± SE (*n* = 3); ***p < 0.001

To examine the role of anillin in breast cancer invasion and metastasis, we initially analyzed its expression in a panel of breast cancer cell lines with varying metastatic potential. Expression of anillin was significantly increased in invasive breast cancer cells, such as MDA-MB-231, MDA-MB-468, BT549, and Hs578t, as compared to the weakly invasive MCF10A, MCF7, and BT474 cells (Additional file 2: Figure S1A,B). Furthermore, this protein was expressed at a higher level in an invasive transformed MCF10A1d cell line compared to parental MCF10A cells and the less invasive, transformed MCF10AneoT cells (Additional file 2: Figure S1C,D). These results indicate that high anillin expression positively correlates with the metastatic potential of breast cancer cells. Next, CRISPR/Cas9 technology was used to deplete anillin in highly invasive breast cancer cells. Stable anillin-deficient cell lines were created using lentiviral transduction of four different anillin-specific single-guide (sg) RNAs. A lentivirus bearing scrambled sgRNA was used to make control cell lines. Puromycin-selected anillin-depleted MDA-MB-231 cell lines demonstrated very efficient (more than 90%) downregulation of this protein (Fig. 1a). Importantly, loss of anillin expression dramatically attenuated both collective migration of MDA-MB-231 cells during wound closure (Fig. 1b,c) and their invasion into Matrigel (Fig. 1d,e). Similar attenuation of wound healing and matrix invasion was observed in anillin-depleted BT549 cells (data not shown). As a complementary approach, anillin was overexpressed in poorly invasive MCF10AneoT cells by using CRISPR/Cas9-dependent activation of its endogenous promoter, which resulted in an approximately eightfold increase in the protein expression (Fig. 2a). Such anillin overexpression significantly accelerated wound healing (Fig. 2b,c) and promoted the Matrigel invasion of MCF10AneoT cells (Fig. 2d,e). Anillin was previously implicated in the regulation of cell proliferation, which can be a contributing factor for altered cell motility. Therefore, we next sought to determine whether altered proliferation plays a role in the anillin-dependent modulation of breast cancer cell migration. Surprisingly, neither knockout nor overexpression of anillin affected the proliferation of breast cancer cells cultured on plastic (Fig. 3a,b). By contrast, loss of anillin dramatically attenuated colony formation of MDA-MB-231 cells cultured on soft agar (Fig. 3c,d), whereas anillin overexpression markedly stimulated soft agar growth of MCF10AneoT cells (Fig. 3e,f). In our early attempts to create anillin-overexpressing breast cancer cells, we transduced MCF10AneoT cells with a retroviral construct encoding an *N*-terminally GFP-tagged anillin. Surprisingly, the stable cell line obtained contained a rapidly truncated overexpressed protein and retained a ~ 40 kDa N-terminal fragment of anillin with the GFP tag (Additional file 3: Figure S2A). The identity of the isolated fragment was further confirmed by mass spectroscopy (data not shown). Interestingly, overexpression of such truncated anillin accelerated Matrigel invasion and soft agar growth of MCF10AneoT cells (Additional file 3: Figure S2B,C), indicating that the N-terminal part of the molecule is essential for the tumor-promoting activities of anillin in breast cancer cells in vitro.

### Loss of anillin expression attenuates breast cancer growth and metastasis in vivo

**Fig. 4:**
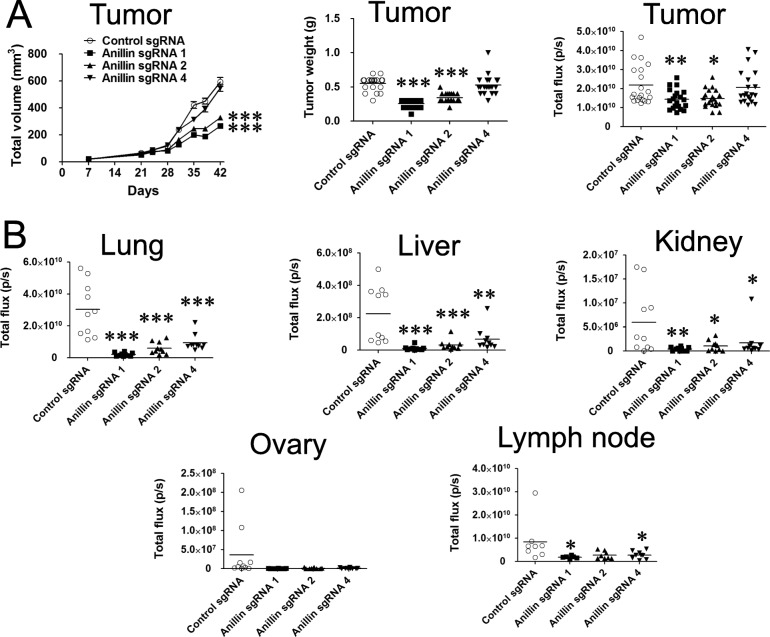
Loss of anillin attenuates primary breast tumor growth and inhibits tumor metastasis in vivo. Control and three different anillin knockout MDA-MB-231 cell lines stable-expressing a luciferase construct were injected into the mammary gland of NSG mice. **a** Tumor volume was measured starting at day 7 after injection of the cells, whereas weight and total luciferase intensity of dissected tumors was measured 6 weeks after the injection at the end of the experiment. **b** Luciferase intensity in isolated liver, lungs, kidney, ovary, and lymph nodes was measured by IVIS at the end of the experiment. Data is presented as mean ± SE (*n* = 10–11); *p < 0.05; **p < 0.01; ***p < 0.001

To demonstrate the pathophysiologic relevance of our in vitro findings, we examined the effects of anillin on breast cancer growth and metastasis in vivo. Control and anillin-deficient MDA-MB-231 cells were infected with a lentivirus-containing tdTomato fluorescent protein and luciferase, and stable cell lines constitutively co-expressing these fluorescent and luminescent reporters were sorted for high tdTomato expression by flow cytometry. To assess the effects of anillin on tumor growth and spontaneous metastases from the primary site, sorted MDA-MB-231 cells were injected into the lactiferous duct of the fourth mammary gland of NSG mice. Primary tumor growth of the injected cells was significantly inhibited by knockout of anillin with sgRNAs 1, 2 (Fig. 4a), and 3 (Additional file 4: Figure S3A,B). Surprisingly, anillin depletion by sgRNA 4 did not affect primary tumor growth (Fig. 4a), despite inhibiting in vitro soft agar growth and motility similarly to other anillin sgRNAs (Figs. 1 and 3). Immunohistologic analysis of dissected primary tumors revealed that cell proliferation (measured by % of cells expressing Ki-67) was not altered in the anillin-depleted MDA-MB-231-derived tumors compared to control tumors (Additional file 5: Figure S4A,B). However, anillin depletion with sgRNA 2 significantly increased the number of cleaved caspase-3-positive cells in the tumors, whereas sgRNA 1-mediated anillin depletion showed a tendency for cleaved caspase increase, thereby indicating accelerated cell apoptosis (Additional file 5: Figure S4C,D). Interestingly, both primary tumor-suppressing sgRNA 1 and sgRNA 2, as well as sgRNA 4 (that did not affect primary tumor growth), attenuated metastatic dissemination of MDA-MB-231 cells into the lungs, liver, kidney, and lymph nodes from the mammary gland (Fig. 4b). This suggests the independent effects of anillin on primary growth and metastasis of breast cancer in vivo. To further confirm specific roles of anillin in metastatic dissemination of breast cancer cells, we used a primary tumor-independent model of tumor metastasis involving breast cancer cell injection into the left ventricle of the mouse heart. Intracardiac injection of anillin sgRNA 1- and sgRNA 4-depleted MDA-MB-231 cells inhibited their dissemination throughout the body and significantly attenuated metastasis in the liver (Additional file 6: Figure S5). Additionally, metastasis of anillin sgRNA 1-depleted cells into the lungs, kidney, ovary, and bones was significantly inhibited as compared to the control cells (Additional file 6: Figure S5). Anillin sgRNA 4-depleted cells showed a tendency for lower metastasis into these organs; however, the effects did not reach the statistical significance (Additional file 6: Figure S5). In support of this in vivo data, overexpression of anillin (truncated N-terminal fragment) in MCF10AneoT cells significantly accelerated primary tumor growth comparing to GFP-expressing controls (Additional file 7: Figure S6A). Furthermore, breast cancer cells with truncated anillin overexpression showed a tendency to have increased metastases in the lungs (Additional file 7: Figure S6B). Overall, these data highlight anillin as an important driver of breast cancer growth and metastasis in vivo.

### Anillin depletion alters extracellular matrix adhesion and the cytoskeletal architecture of breast cancer cells

**Fig. 5:**
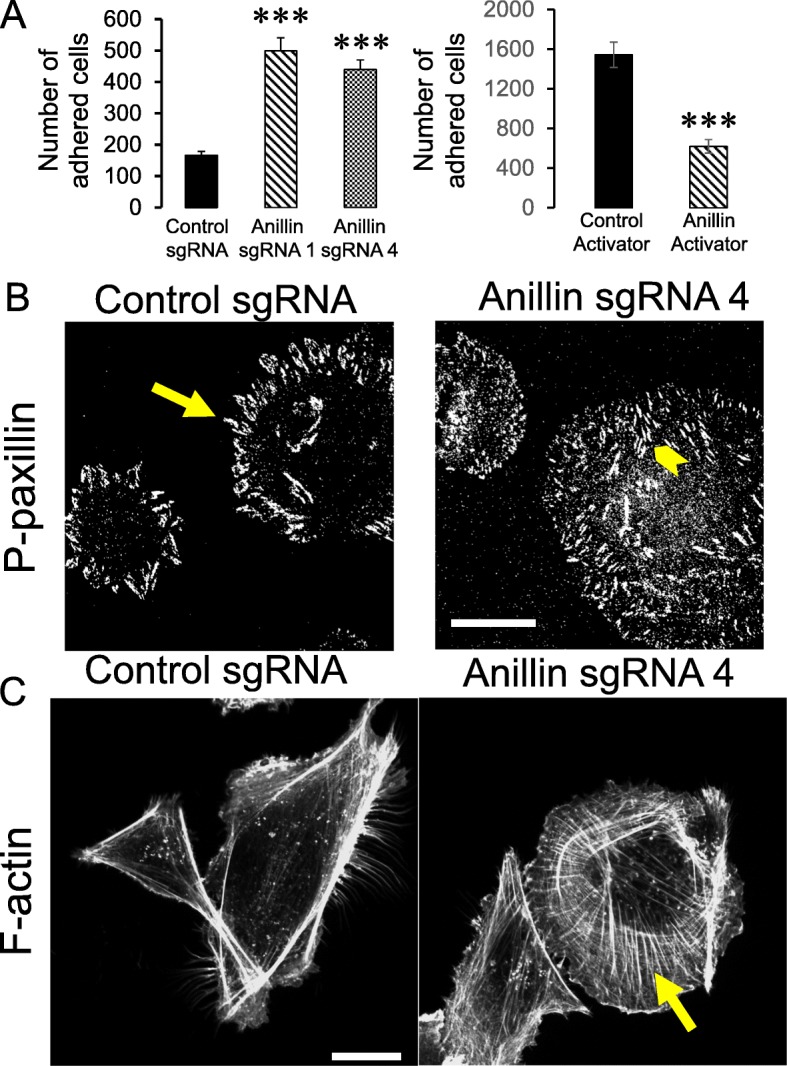
Anillin regulates cell-ECM adhesion and organization of the actin cytoskeleton. **a** Control and anillin-depleted MDA-MB-231 cells, as well as control and anillin-overexpressing MCF10AneoT cells, were plated on collagen I-coated dishes, and the adherent cell number was determined after 1 h of plating. **b**,**c** Control and anillin-depleted MDA-231 cells were fixed and fluorescently labeled for either a FA marker, phosphorylated (P) paxillin (**b**), or filamentous (F) actin (**c**). Arrows point on FA localization at the edges of control MDA-231 cells (**b**), or show the assembly of basal stress fibers in anillin-depleted cells (**c**). Arrowhead highlights FA located throughout the entire basal surface of anillin-depleted cells (**b**). Data is presented as mean ± SE (*n* = 3); ***p < 0.001. Scale bars, 20 μm

Next, we sought to elucidate the mechanisms that underlie anillin-dependent effects on breast cancer cell migration and invasion. Since cell motility depends on the interactions with extracellular matrix (ECM), we examined whether anillin expression modulates ECM adhesion of breast cancer cells. Loss of anillin in MDA-MB-231 cells markedly increased their adhesion to collagen I, whereas overexpression of full-length anillin in MCF10AneoT cells inhibited collagen adhesion (Fig. 5a). The described effects of anillin on ECM adhesion were recapitulated when collagen I was replaced with fibronectin (data not shown), while neither control nor anillin-depleted MDA-MB-231 cells have shown strong adhesion to collagen IV and laminin (data not shown). Cell attachment to ECM is known to be regulated by focal adhesion (FA) complexes formed at the interface between the plasma membrane and the substratum. Therefore, we next examined if altered FA assembly mediates hyper adhesiveness of anillin-depleted cells, by performing immunolabeling of a classical FA maker, phosphorylated (P) paxillin. Control MDA-MB-231 cells demonstrated prominent P-paxillin labeling at FA that was confined to the edges of spreading cells (Fig. 5b, arrow). By contrast, loss of anillin caused the distribution of FA throughout the entire basal surface of the cells (Fig. 5b, arrowhead). Interestingly, immunoblotting analysis did not show significant differences in the expression of total or phosphorylated paxillin, focal adhesion kinase, and Src kinase, as well as any differences in α and β integrin subunit expression between control and anillin-depleted MDA-MB-231 cells (Additional file 8: Figure S7). This suggests that loss of anillin does not directly affect the FA signaling, but rather alters the cellular topography of these adhesion complexes. Since cellular distribution of FA depends on their association with the actomyosin cytoskeleton and anillin is a known actin and NM II-binding protein, we rationalized that anillin could regulate FA localization indirectly, by modulating organization of the FA-associated cytoskeleton. Indeed, fluorescence F-actin labeling and confocal microscopy revealed dramatic alterations in the cytoskeletal architecture in anillin-depleted MDA-MB-231 cells, manifested by the assembly of prominent basal stress fibers (Fig. 5c, arrow). Similar stress fiber formation was observed in our previous study involving anillin depletion in prostate epithelial cells [25]. Given a crucial role of the actin motor, NM II, in regulating stress fiber assembly and cell migration [45], we sought to investigate the involvement of NM II in the impaired motility of anillin-depleted breast cancer cells. Inhibition of NM II activity using a specific pharmacological inhibitor, blebbistatin (50 μM), inhibited the assembly of basal stress fibers (Additional file 9: Figure S8A) and reversed attenuated wound healing of anillin-depleted MDA-MB-231 cells (Additional file 9: Figure S8B), thereby highlighting the involvement of the actomyosin cytoskeleton. Finally, we sought to investigate the mechanisms of the cytoskeletal remodeling in anillin-depleted cancer cells. Given the known roles of anillin in controlling the activity of Rho family of small GTPases [16, 18–20], we compared the activation status of RhoA and Rac1 in control and anillin-depleted MDA-MB-231 cells. No significant effect of anillin knockout on the amount of active RhoA and Rac1 was observed (Additional file 10: Figure S9).

Based on the published data [13, 14], we also rationalized that anillin can affect the cytoskeletal organization in breast cancer cells by its direct binding to either actin filaments, or the NM II motor. However, immunofluorescence labeling of anillin revealed its predominantly nuclear location in non-dividing MDA-MB-231 cells and a lack of colocalization with cytoplasmic actin filaments (Additional file 11: Figure S10). These data are consistent with previously published studies documented mostly a nuclear accumulation of anillin in different cancer cells [25, 28]. Together, these results do not support the idea that anillin acts as a peripheral scaffolding protein, directly affecting different cytoplasmic or cortical cytoskeletal structures in breast cancer cells. Instead, anillin is likely to control the peripheral cytoskeleton, ECM adhesion, cell motility, and growth indirectly, by functioning as a nuclear regulator of the cellular homeostasis.

### Modulation of anillin expression affects breast cancer cell stemness and differentiation

**Fig. 6:**
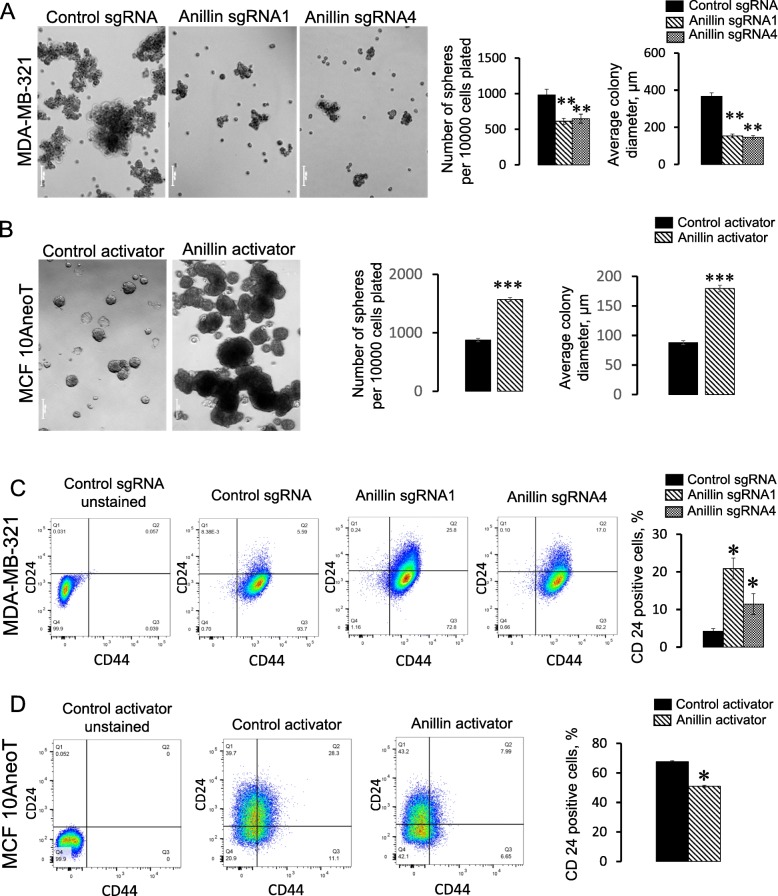
Anillin regulates the progenitor/stem cell properties of breast cancer cells. Control and anillin-depleted MDA-MB-231 cells (**a**), as well as control and anillin-overexpressing MCF10AneoT cells (**b**) were cultured in suspension, in stem cell supporting Mammocult medium. Cells were photographed and the number of mammospheres were counted on day 7 after plating. Control, anillin-deficient (**c**), and anillin-overexpressing (**d**) cells were dual-immunolabeled with antibodies against CD24 and CD44 and the labeled cells were subjected to a flow cytometry analysis. Data is presented as mean ± SE (*n* = 3); *p < 0.05; **p < 0.01; ***p < 0.001

The idea that anillin may have global regulatory effects on breast cancer cell homeostasis is consistent with the aforementioned results about breast tumor growth and proliferation (Fig. 3). Thus, the specific effect of anillin expression on the anchorage-independent, versus the anchorage-dependent growth of breast cancer cells suggests that it may regulate breast cancer stem cells (BCSC). Therefore, we sought to directly examine the roles of anillin in BCSC regulation by using two different experimental approaches. One approach was a classical mammosphere formation assay to determine a self-renewal capacity of BCSC, whereas the other was flow cytometry experiments to quantify the expression of stem cell-specific surface markers [46–48]. We observed that loss of anillin expression significantly attenuated mammosphere growth in MDA-MB-231 (Fig. 6a) and BT549 (data not shown) cells, whereas the increased anillin expression promoted mammosphere formation in MCF10AneoT cells (Fig. 6b). Flow cytometry analysis of control MDA-MB-231 cells revealed a high prevalence of CD44^high^/CD24^low^ cells (Fig. 6c), which is consistent with previously reported BCSC-like features of MDA-MB-231 cells [49]. Anillin depletion markedly increased a fraction of CD24^high^ cells (Fig. 6c), indicating a shift toward cell differentiation. Consistently, anillin overexpression significantly decreased the population of CD24^high^ MCF10AneoT cells (Fig. 6d). Collectively, these data suggest that high anillin level stimulates BCSC, whereas loss of anillin inhibits breast cancer cell stemness.

**Fig. 7:**
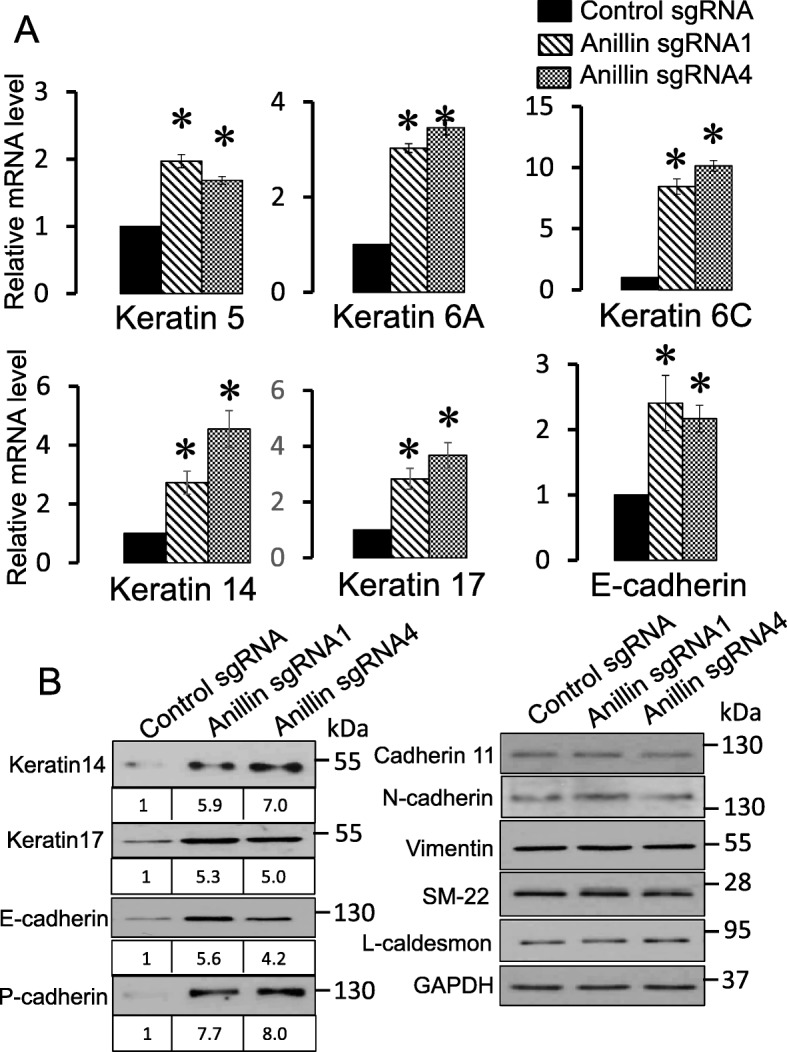
Anillin knockout upregulates expression of basal epithelial cell markers. **a** Quantitative RT-PCR analysis of keratins and E-cadherin expression in control and anillin-depleted MDA-231 cells. **b** Immunoblotting analysis of different epithelial and mesenchymal markers in control and anillin-depleted MDA-MB-231 cells. Data is presented as mean ± SE (*n* = 3); *p < 0.05

To investigate if the decreased stemness of anillin-deficient cells is associated with their transcriptional reprogramming, we performed RNA sequencing analysis (RNAseq) of control and anillin-depleted MDA-MB-231 cell lines. This analysis showed profound alterations in gene expression in anillin-depleted cells with a number of significantly up- and downregulated genes (Additional files 12 and 13: Tables S2 and S3). A gene ontology analysis of the RNAseq data revealed that loss of anillin caused upregulation of molecular pathways associated with epithelial and epidermal differentiation and downregulation a number of genes associated with RNA polymerase II (RNAPII)-dependent gene transcription (Additional file 14: Figure S11). Interestingly, several low molecular weight keratins, including keratins 5, 6, 14, and 17, were among the highest upregulated genes in anillin-depleted cells according to the RNAseq data (Additional file 12: Table S2). We confirmed the upregulation of these keratins by quantitative RT-PCR and immunoblotting in MDA-MB-231 (Fig. 7) and BT549 (data not shown) cells. Keratins 5, 6, 14, and 17 are known markers of the basal-type breast cancer [50, 51]. Induction of these keratins suggests that loss of anillin results in the basal-type trans-differentiation of mesenchymal breast cancer cells, such as MDA-MB-231 cells. If this suggestion is correct, anillin depletion should induce the expression of other basal epithelial cell markers, including epithelial cadherins. Indeed, we found significant upregulation of E-cadherin and P-cadherin expression in anillin-depleted MDA-MB-231 cells, both at the mRNA and protein levels, which was not accompanied by altered expression of mesenchymal markers (Fig. 7). Furthermore, increased E-cadherin expression was observed in MDA-MB-231-derived breast tumors in vivo (Additional file 5: Figure S4E,F). Surprisingly, anillin overexpression did not decrease protein levels of E-cadherin, P-cadherin, and keratin 17 in MCF10AneoT cells (data not shown), indicating that high anillin expression alone is not sufficient to trigger significant de-differentiation of epithelial-type breast cancer cells. Our RNAseq analysis also provides some clues regarding the mechanisms that could mediate anillin-dependent effects on breast cancer cell stemness and differentiation. Thus, anillin-deficient MDA-MB-231 cells showed significant expressional downregulation of several transcription factors, such as TBX18, OVOL2, TFCP2L1, FOXK1, PBX1, HCFC1, and Sox-9, which are known to regulate cell stemness and differentiation (Additional file 13: Table S3). Attenuated expression of these transcription factors is consistent with the observed decrease in the self-renewal potential of breast cancer cells caused by anillin knockout (Fig. 6).

### Downregulation of E-cadherin reverses attenuated migrating and invasion of anillin-depleted breast cancer cells

**Fig. 8:**
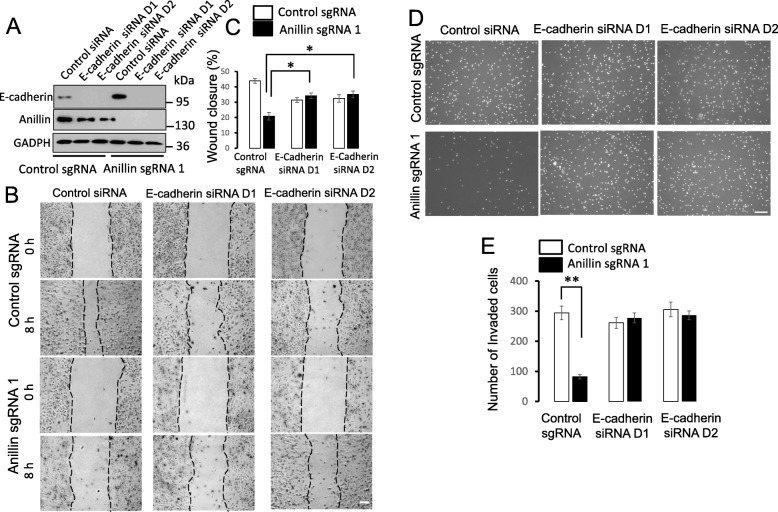
Downregulation of E-cadherin expression reverses the decreased motility of anillin-deficient breast cancer cells. E-cadherin was transiently depleted in control and anillin-deficient MDA-MB-231 cells by two different siRNAs. **a** Immunoblotting analysis shows the efficiency of E-cadherin depletion. **b**,**c** Representative images and quantification of wound healing in control and anillin-deficient cell monolayers with and without E-cadherin depletion. Scale bar, 100 μm. **d**,**e** Representative images and quantification analysis of Matrigel invasion of control and anillin-overexpressing MDA-MB-231 cells transfected with either control or E-cadherin-specific siRNAs. Data are presented as mean ± SE (*n* = 3); *p < 0.05; **p < 0.001. Scale bar, 50 μm

Finally, we sought to investigate if the observed trans-differentiation of anillin-depleted MDA-MB-231 cells plays a causal role in inhibiting cell migration and invasion. We focused on cadherin overexpression since both E- and P-cadherins are known regulators of cell migration [52, 53]. Either E-cadherin or P-cadherin were transiently downregulated in control and anillin-depleted MDA-MB-231 cells and effects of their depletion on cell motility were determined using the wound closure and Matrigel invasion assays. E-cadherin depletion decreased wound healing of control cells and modestly increased wound healing of anillin-depleted cells, thereby eliminating the inhibitory effect of anillin depletion on the collective cell migration (Fig. 8a–c). Furthermore, the downregulation of E-cadherin reversed the attenuated invasion of anillin-depleted cells, while not altering control cell invasion (Fig. 8d,e). By contrast, downregulation of P-cadherin expression decreased wound healing and invasion of control MDA-MB-231 cells, but did not affect the motility of anillin-depleted cells (Additional file 15: Figure S12). Together, these results demonstrate that the upregulation of E-cadherin expression plays essential roles in the attenuated migration and invasion of breast cancer cells caused by anillin knockout.

## Discussion

Anillin is a unique cytoskeletal scaffolding protein, which is markedly upregulated in many solid tumors [26–32]. Although it is known to control various processes in mitotic and non-dividing cells, the roles of anillin in oncogenesis remain poorly investigated. In the present study, we show that anillin has a dual function in breast cancer development by promoting growth and metastatic dissemination of cancer cells in vitro and in vivo. These pro-motile and growth-promoting functions of anillin appear to be independent. Indeed, anillin acted as an essential driver of collective migration and matrix invasion of breast cancer cells in vitro without affecting their anchorage-dependent proliferation (Figs. 1, 2 and 3). Furthermore, loss of anillin attenuated the metastatic dissemination of breast cancer cells in vivo even when it did not inhibit the primary tumor growth (Fig. 4). Lack of the effects of anillin knockout and overexpression on proliferation of adherent breast cancer cells is surprising, given the previous studies that document proliferation/cell cycle defects in different mammalian cell lines after anillin depletion by RNA interference [28, 32, 33]. There are several reasons for this discrepancy. One is the possible contribution of nonspecific cell growth-restricting effects of anillin siRNAs, driven by the disruption of endogenous miRNA functions [54]. Alternatively, it is possible that stable CRISPR/Cas9-edited breast cancer cell lines are capable of compensating for some, but not all, functional defects caused by the anillin knockout. The existence of such compensatory mechanisms is illustrated by the anillin-deficient MDA-MB-231 cell line created with the sgRNA 4, which unlike other derived cell lines, displayed unaffected primary tumor growth despite the dramatic loss of anillin expression (Fig. 4). Interestingly, even suppressed in vivo growth of anillin-depleted primary tumors was not due to inhibition of tumor cell proliferation, given similar percentage of Ki-67-positive cells detected in anillin-depleted and control MDA-MB-231-derived tumors (Additional file 5: Figure S4A,B). It is likely that such tumor growth inhibition could be mediated by a combination of different mechanisms that include the increased cell apoptosis (Additional file 5: Figure S4C,D), inefficient cytokinesis and other yet to be defined abnormalities of anillin-depleted breast cancer cells. Overall, our findings of the anillin-dependent regulation of anchorage-independent breast cancer cell growth in vitro (Fig. 3c–f) and breast tumor xenograft development in vivo (Fig. 4a and Additional file 4: Figure S3) highlight the essential tumor growth-promoting functions of this protein.

Our study also revealed a non-canonical mechanism that mediates anillin activity in breast cancer cells. This mechanism appears to be independent of the direct anillin interactions with peripheral cytoskeletal structures and involves transcriptional reprogramming of breast cancer cells affecting their stemness and differentiation. Generally, the sites of anillin actions in non-dividing epithelial and cancer cells remain controversial. Thus, this protein was shown to be enriched at intercellular junctions in *Xenopus* embryos, where it locally controlled RhoA activity and organization of the cortical actin cytoskeleton [18, 23]. Furthermore, peripheral anillin was found to regulate podosome assembly in specialized mammalian cells, such as podocytes [24]. By contrast in prostate and colon cancer cells, anillin is confined to the nucleus and remotely regulates the architecture of the cortical actin cytoskeleton and intercellular junctions by modulating c-Jun terminal kinase signaling [25]. Furthermore, studies of the transgenic mouse expressing EGFP-anillin reporter report predominantly nuclear localization of this protein in non-dividing cells of different tissues in vivo [55, 56]. In agreement with these studies and previously described immunolabeling of breast cancer tissues [28], we found predominantly nuclear accumulation of anillin in breast cancer cells (Additional file 11: Figure S10). Despite such nuclear localization, anillin was able to control the molecular events in the cytoplasm and the cortex of cancer cells, including assembly of the actin cytoskeleton and distribution of FA (Fig. 5).

One of the most striking findings of the present study is a novel role of anillin as a potent positive regulator of BCSC. Upregulation of anillin expression markedly enhanced the self-renewal potential of MCF10AneoT cells, whereas loss of anillin decreased stem/progenitor properties of MDA-MB-231 and BT549 cells (Fig. 6 and data not shown). Interestingly, anillin depletion induced a molecular signature consistent with trans-differentiation of these mesenchymal breast cancer cells into basal-like epithelial cells (Fig. 7), although anillin overexpression failed to decrease the levels of epithelial markers in MCF10AneoT cells (data not shown). These results indicate that anillin predominantly acts as a regulator of the phenotypic plasticity in poorly differentiated cancer cells and stem cells, being much less efficient in perturbing the phenotype of well-differentiated epithelial cells. The described modulation of stemness and differentiation of breast cancer cells can explain key functional effects of anillin knockout and overexpression, such as modulation of anchorage-independent cell growth in vitro and tumor xenograft development in vivo (Figs. 3 and 4). Furthermore, the mesenchymal-epithelial differentiation is likely to be responsible for the suppressed motility of anillin-depleted breast cancer cells. One of the key events of such trans-differentiation is expressional upregulation of E-cadherin (Fig. 7; Additional file 5: Figure S4E,F), which is a known suppressor of cancer cell migration and metastasis [52]. Consistently, our experiments demonstrated a causal role for E-cadherin upregulation in the attenuated motility of anillin-depleted breast cancer cells in vitro (Fig. 8). The upregulation of E-cadherin may not explain all functional effects of the anillin knockout, and it likely acts together with other mechanisms. These mechanisms may involve the reorganization of the actomyosin cytoskeleton since our data demonstrate a robust assembly of basal F-actin stress fibers in anillin-depleted breast cancer cells (Fig. 5c) and a complete reversal of the attenuated wound healing of anillin-depleted cells by NM II inhibition (Additional file 9: Figure S8).

While anillin has not been recognized as an important regulator of cancer stem cells, some previous reports linked this protein to stem cell biogenesis. For example, anillin was found to be enriched in rapidly proliferating mammalian pluripotent stem cells [56] and was repressed in the senescent cervical carcinoma cells [57]. Furthermore, anillin was shown to control the fate of neuronal progenitor cells in zebrafish retina [58]. Interestingly, our RNAseq data revealed downregulation of several transcription factors essential for stem cell biogenesis, including TBX18, OVOL2, TFCP2L1, FOXK1, PBX1, HCFC1, and Sox-9 in anillin-depleted MDA-MB-231 cells (Additional file 13: Table S3). Future studies are needed to understand the roles and mechanisms of the anillin-dependent regulation of cancer stem cells.

Our RNAseq analysis indicates that inhibition of stemness and epithelial trans-differentiation is associated with a global transcriptional reprogramming of anillin-depleted breast cancer cells (Additional file 14: Figure S11; Additional files 12 and 13: Tables S2 and S3). This is consistent with recent studies showing global alterations in gene expression in anillin-depleted pancreatic adenocarcinoma and bladder urothelial carcinoma cells [27, 32]. While the mechanisms of anillin-dependent regulation of gene expression remain unknown, they could be associated with control of polymerization and functions of nuclear actin. Indeed, nuclear actin is a well-known regulator of gene transcription that binds to and modulates the activity of RNAPII and other important transcriptional regulators [59–61]. Several studies implicated nuclear actin in the regulation of stemness and differentiation of different cells [62–64]. On the other hand, anillin is a well-known actin-binding protein accumulated in the nuclei of breast cancer cells (Additional file 11: Figure S10). Furthermore, the truncated N-terminal anillin fragment that promoted MCF10AneoT cell growth and migration (Additional file 3: Figure S2; Additional file 7: Figure S6) displayed nuclear localization (data not shown) and most likely retained the NM II and actin-binding sites located in the N-terminal region of the anillin molecule [12]. We hypothesize that anillin interacts with nuclear actin and modulates its binding to essential components of the transcriptional machinery such as RNAPII. This results in altered expression of the subsets of genes that regulate breast cancer stemness and differentiation. Our RNAseq analysis provides support for this idea by showing downregulation of the pathways involving RNAPII-dependent transcription in anillin-deficient MDA-MB-231 cells (Additional file 14: Figure S11; Additional file 13: Table S3). Further studies will clarify a possible functional interplay between anillin and nuclear actin in the regulation of phenotypic plasticity and functions of different cancer cells.

## Conclusion

In conclusion, our study revealed that a cytoskeletal scaffolding protein anillin drives growth and metastatic dissemination of cancer cells in vitro and in vivo. The mechanisms of such tumor-promoting activity involve transcriptional reprogramming of breast cancer cells affecting their self-renewal properties and differentiation. Given a marked overexpression of anillin in breast cancer and other solid tumors, this cytoskeletal protein appears to be an attractive target for developing tumor-suppressing and antimetastatic therapies.

## Supplementary information

Additional file 1: **Table S1.** (A) Sequences of the single guide (sg) RNA used for CRISPR/Cas9-dependent anillin knockout. (B) Primer sequences for quantitative RT-PCR analysis of the selected genes in control and anillin deficient MDA-MB-231 cells.

Additional file 2: **Figure S1.** Anillin is highly expressed in invasive breast cancer cell lines. Anillin expression was determined in a panel of poorly and highly-invasive breast cancer cell lines (A,B) and a series of MCF10A-derived breast cancer cells with different invasiveness (C,D). Data are presented as mean ± SE (*n* = 3); *p < 0.05; **p < 0.01; ***p < 0.001.

Additional file 3: **Figure S2.** Overexpression of the truncated anillin mutant stimulates breast cancer cell invasion and soft agar growth. (A) Immunoblotting shows the expression of a full-length GFP-anillin at an early passage and the appearance of truncated GFP-labeled anillin fragment in a late passage of MCF10AneoT cells stably transfected with GFP-anillin. (B) Matrigel invasion and (C) soft agar growth of control and truncated anillin fragment (tAnillin)-overexpressing MCF10AneoT cells. Data are presented as mean ± SE (n = 3); **p < 0.01; ***p < 0.001.

Additional file 4: **Figure S3.** Anillin depletion with sgRNA 3 inhibits growth of primary breast tumor in vivo. Control and anillin-sgRNA 3-depleted MDA-MB-231 cells were injected into the mammary gland of NSG mice. Tumor volume was measured starting at day 14 after injection of the cells (A), whereas weight of the dissected tumors was measured at the end of the experiment (B). Data is presented as mean ± SE (*n* = 12–14); **p < 0.01; ***p < 0.001.

Additional file 5: **Figure S4.** Anillin depletion increases E-cadherin and cleaved caspase-3 expression in MDA-MB-231cell-derived breast tumors in vivo. Control and two different anillin-knockout MDA-MB-231 cell lines (Anillin sgRNA 1 and sgRNA 2) were injected into the mammary gland of NSG mice. Ki-67, cleaved caspase-3, and E-Cadherin levels were examined by IHC staining of the mammary tumors. The staining was imaged with Vectra Polaris (A,C,E) and quantified (B,D,F) using Inform software. Data are presented as mean ± SE (*n* = 4); **p < 0.001; ****p < 0.0001. Arrows, cleaved caspase-3 and E-cadherin staining.

Additional file 6: **Figure S5.** Loss of anillin inhibits breast cancer cell metastasis in vivo. Control and anillin sgRNA1 and sgRNA4-depleted MDA-MB-231 cell lines stable-expressing a luciferase construct were injected intracardially in NSG mice. Luciferase intensity of the dorsal and ventral side of the mouse was monitored starting on 7 days after injection. Four weeks after the injection, luciferase intensity of isolated lungs, liver, kidney, ovary, bones and brain was measured by IVIS. Data is presented as mean ± SE (n = 12–14); *p < 0.05; **p < 0.01; ***p < 0.001.

Additional File 7: **Figure S6.** Truncated anillin fragment stimulates primary breast tumor growth in vivo. MCF10AneoT cells stably expressing either truncated anillin-GFP (tAnillin) or control GFP along with a luciferase construct were injected into the mammary gland of NSG mice. (A) Luciferase intensity at the ventral side of the mouse and tumor volume were measured starting at days 20 and 42 after injection of the cells, respectively, whereas volume, weight and total luciferase intensity of dissected tumors was measured ten weeks after the injection at the end of the experiment. (B) Luciferase intensity in isolated lungs, liver, ovary, and kidney, was measured by IVIS at the end of the experiment. Data is presented as mean ± SE (*n* = 10–11); **p < 0.01; ***p < 0.001.

Additional file 8: **Figure S7.** Anillin depletion does not affect expression and activation of ECM adhesion proteins. Immunoblotting analysis of focal adhesion proteins and integrin subunits expression in control and anillin-depleted MDA-MB-231 cells.

Additional file 9: **Figure S8.** Inhibition of NM II reverses attenuated collective migration of anillin-depleted cells. Control and anillin-knockout MDA-MB-231 cells were incubated with either vehicle, or a NM II inhibitor, blebbistatin (50 μM). (A) The effect of blebbistatin on the actin cytoskeleton architecture was determined by phalloidin labeling and confocal microscopy. Arrow indicates stress fibers in vehicle treated anillin-depleted cells, whereas arrowhead points on disappearance of stress fibers in blebbistatin-exposed anillin-depleted cells. (B) The effects of blebbistain on collective cell migration was examined during 12 h wound closure assay. Data is presented as mean ± SE (*n* = 3); **p < 0.01. Scale bar, 20 μm.

Additional file 10: **Figure S9.** Anillin depletion does not alter the activity of RhoA and Rac1 small GTPases. The amount of active RhoA in control and anillin-depleted MDA-MB-231 cells was determined by a specific G-LISA assay (A), whereas Rac1 activity was examined by a pull-down of active Rac1 with subsequent immunoblotting analysis of active and total Rac1 expression (B).

Additional file 11: **Figure S10.** Nuclear localization of anillin is the in invasive breast cancer cells. (A) Parental MDA-MB-231 cell were immunofluorescence labeled for anillin (green), whereas DAPI (blue) was used to label nuclei. (B) Control and anillin-depleted MDA-MB-231 cells were dual-immunolabeled for anillin (green) and F-actin (red). Arrow point on nuclear localization of anillin that disappeared in anillin-deficient cells. Scale bar, 20 μm.

Additional file 12: **Table S2.** List of genes upregulated in anillin-deficient MDA-MB-231 cell lines as compared to control cells. “Geneid”, “symbol”, “description” - gene annotations; “logFC.1”, “logFC.2” - log fold change in the first and second CRISPR experiments, respectively; “Average logFC” – average log fold change; “FDR.1”, “FDR.2” - FDR-adjusted *p*-value of differential expression in the first and second CRISPR experiments, respectively.

Additional file 13: **Table S3.** List of genes downregulated in anillin-deficient MDA-MB-231 cell lines as compared to control cells. “Geneid”, “symbol”, “description” - gene annotations; “logFC.1”, “logFC.2” - log fold change in the first and second CRISPR experiments, respectively; “Average logFC” – average log fold change; “FDR.1”, “FDR.2” - FDR-adjusted p-value of differential expression in the first and second CRISPR experiments, respectively.

Additional file 14: **Figure S11.** Loss of anillin causes transcriptional reprogramming of breast cancer cells. RNA sequencing analysis was performed on mRNA samples isolated from control and anillin-depleted (sgRNA 3 and sgRNA 4) MDA-MB-231 cells. Gene Set enrichment analysis was performed on 275 differentially expressed genes common for two CRISPR sgRNA-derived cell lines. Red and blue bars depict cellular processes upregulated and downregulated in anillin-deficient cells, respectively.

Additional file 15: **Figure S12.** Downregulation of P-cadherin expression does not reverse the decreased motility of anillin-deficient breast cancer cells. P-cadherin was transiently depleted in control and anillin-deficient MDA-MB-231 cells by a selective siRNA SmartPool. (A) Immunoblotting analysis shows the efficiency of P-cadherin depletion. (B,C) Representative images and quantification of wound healing in control and anillin-deficient cell monolayers with and without P-cadherin depletion. Scale bar, 100 μm. (D,E) Representative images and quantification analysis of Matrigel invasion of control and anillin-overexpressing MDA-MB-231 cells transfected with either control or P-cadherin-specific siRNAs. Data are presented as mean ± SE (n = 3). Scale bar, 50 μm.

## Abbreviations

BCSC: Breast cancer stem cells
CRISPR: Clustered Regularly Interspaced Short Palindromic Repeats
Cas: CRISPR-associated
ECM: Extracellular matrix
FA: Focal adhesions
IHC: Immunohistochemistry
IVIS: In Vivo Imaging System
NM II: Non-muscle myosin II
NSG: NOD-SCID-IL2 gamma-receptor null
RNAseq: RNA sequencing
sgRNA: Single-guide RNA
siRNA: Small interfering RNA
