# Supplementary material for: Anillin regulates breast cancer cell migration, growth, and metastasis by non-canonical mechanisms involving control of cell stemness and differentiation
Source: Breast Cancer Res. 2020 Jan 7;22:3. doi: 10.1186/s13058-019-1241-x (PMC6947866; doi:10.1186/s13058-019-1241-x)
Supplement: Supplementary file 1 — Table S1. (A) Sequences of the single guide (sg) RNA used for CRISPR/Cas9-dependent anillin knockout. (B) Primer sequences for quantitative RT-PCR analysis of the selected genes in control and anillin deficient MDA-MB-231 cells. [file 13058_2019_1241_MOESM1_ESM.docx]

**Supplementary Table S1 (A)** Sequences of the single guide (sg) RNA used for CRISPR/CAS9-dependent anillin knockout. (**B**) Primer sequences for quantitative RT-PCR analysis of the selected genes in control and anillin deficient MDA-MB-231 cells.

Supplementary table 1A.

| Gene name | Oligo Name | Direction | Sequence (5'-3') | Resource |
| --- | --- | --- | --- | --- |
| HsANLN | HsANLN-sg1F | Forward | CACCGGAAGATTCTCTCGCCTGGCA | Lander Lab |
|  | HsANLN-sg1R | Reverse | AAACTGCCAGGCGAGAGAATCTTCC |  |
| HsANLN | HsANLN-sg2F | Forward | CACCGGGCGATGGATCCGTTTACGG | Lander Lab |
|  | HsANLN-sg2R | Reverse | AAACCCGTAAACGGATCCATCGCCC |  |
| HsANLN | HsANLN-sg3F | Forward | CACCGAACTCACTCACCTCCGTAAA | Zhang Lab |
|  | HsANLN-sg3R | Reverse | AAACTTTACGGAGGTGAGTGAGTTC |  |
| HsANLN | HsANLN-sg4F | Forward | CACCGTCTCTGAAGATTCTCTCGCC | Zhang Lab |
|  | HsANLN-sg4R | Reverse | AAACGGCGAGAGAATCTTCAGAGAC |  |
| Non-Targeting | Non-Targeting-sgF | Forward | CACCGGACCGGAACGATCTCGCGTA | Lander Lab |
|  | Non-Targeting-sgR | Reverse | AAACTACGCGAGATCGTTCCGGTCC |  |

Supplementary table 1B.

| Gene name | Direction | Sequence (5'-3') |
| --- | --- | --- |
| HsKRT5 | Forward | TGTCACAAGCAGTGTTTCCTCT |
|  | Reverse | ACAAATTTGACGCTGGAGCTG |
| HsKRT6A | Forward | AGTGCAGGTGGGTAACTGAC |
|  | Reverse | CCAGTCAGAAGAGTGCGAGG |
| HsKRT6C | Forward | GCCCCCAAACTCCCTAAGTT |
|  | Reverse | GAGAAGGTTTAAGCACATGGGG |
| HsKRT14 | Forward | AGCCGCCAAATCCGCACCAA |
|  | Reverse | TGATTGCCAGGAGGGGGTGA |
| HsKRT17 | Forward | CACCCGTCAGGTGCGTACCATT |
|  | Reverse | AGGCAAGGAAGCATGGGGAAGG |
| E-cadherin | Forward | TTACTGCCCCCAGAGGATA |
|  | Reverse | TGCAACGTCGTTACGAGTA |
| GAPDH | Forward | CATGTTCGTTCATGGGTGTAACCA |
|  | Reverse | AGTGATGGCATGGACTGTCTCAT |
