# Supplementary material for: Anillin regulates breast cancer cell migration, growth, and metastasis by non-canonical mechanisms involving control of cell stemness and differentiation
Source: Breast Cancer Res. 2020 Jan 7;22:3. doi: 10.1186/s13058-019-1241-x (PMC6947866; doi:10.1186/s13058-019-1241-x)
Supplement: Supplementary file 2 — Figure S1. Anillin is highly expressed in invasive breast cancer cell lines. Anillin expression was determined in a panel of poorly and highly-invasive breast cancer cell lines (A,B) and a series of MCF10A-derived breast cancer cells with different invasiveness (C,D). Data are presented as mean ± SE (n = 3); *p < 0.05; **p < 0.01; ***p < 0.001. [file 13058_2019_1241_MOESM2_ESM.pptx]

## Slide 1
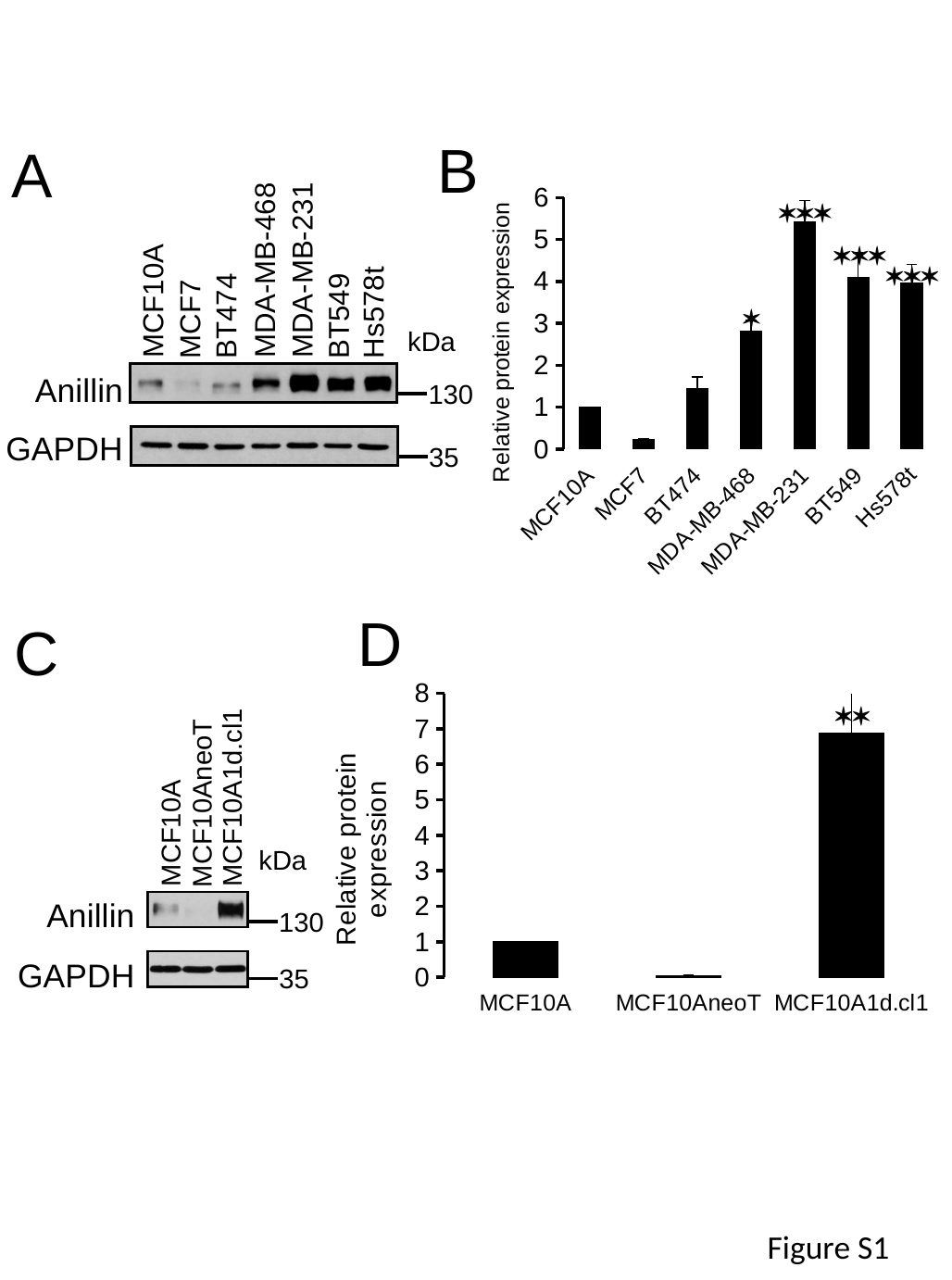

B
A
### Chart
| Category | |
|---|---|
| MCF10A | 1.0 |
| MCF7 | 0.22543691814029929 |
| BT474 | 1.4365329545241508 |
| MDA-MB-468 | 2.8238373620831756 |
| MDA-MB-231 | 5.425848765291473 |
| BT549 | 4.0887505808485445 |
| Hs578t | 3.9715823676361084 |


MDA-MB-468
MDA-MB-231
MCF10A

Hs578t
BT474
BT549
MCF7
kDa
Anillin
130
GAPDH
35
D
C
### Chart
| Category | |
|---|---|
| MCF10A | 1.0 |
| MCF10AneoT | 0.04666666666666666 |
| MCF10A1d.cl1 | 6.88 |
MCF10A1d.cl1
MCF10AneoT
MCF10A
kDa
Anillin
130
GAPDH
35
Figure S1
