# Supplementary material for: Anillin regulates breast cancer cell migration, growth, and metastasis by non-canonical mechanisms involving control of cell stemness and differentiation
Source: Breast Cancer Res. 2020 Jan 7;22:3. doi: 10.1186/s13058-019-1241-x (PMC6947866; doi:10.1186/s13058-019-1241-x)
Supplement: Supplementary file 3 — Figure S2. Overexpression of the truncated anillin mutant stimulates breast cancer cell invasion and soft agar growth. (A) Immunoblotting shows the expression of a full-length GFP-anillin at an early passage and the appearance of truncated GFP-labeled anillin fragment in a late passage of MCF10AneoT cells stably transfected with GFP-anillin. (B) Matrigel invasion and (C) soft agar growth of control and truncated anillin fragment (tAnillin)-overexpressing MCF10AneoT cells. Data are presented as mean ± SE (n = 3); **p < 0.01; ***p < 0.001. [file 13058_2019_1241_MOESM3_ESM.pptx]

## Slide 1
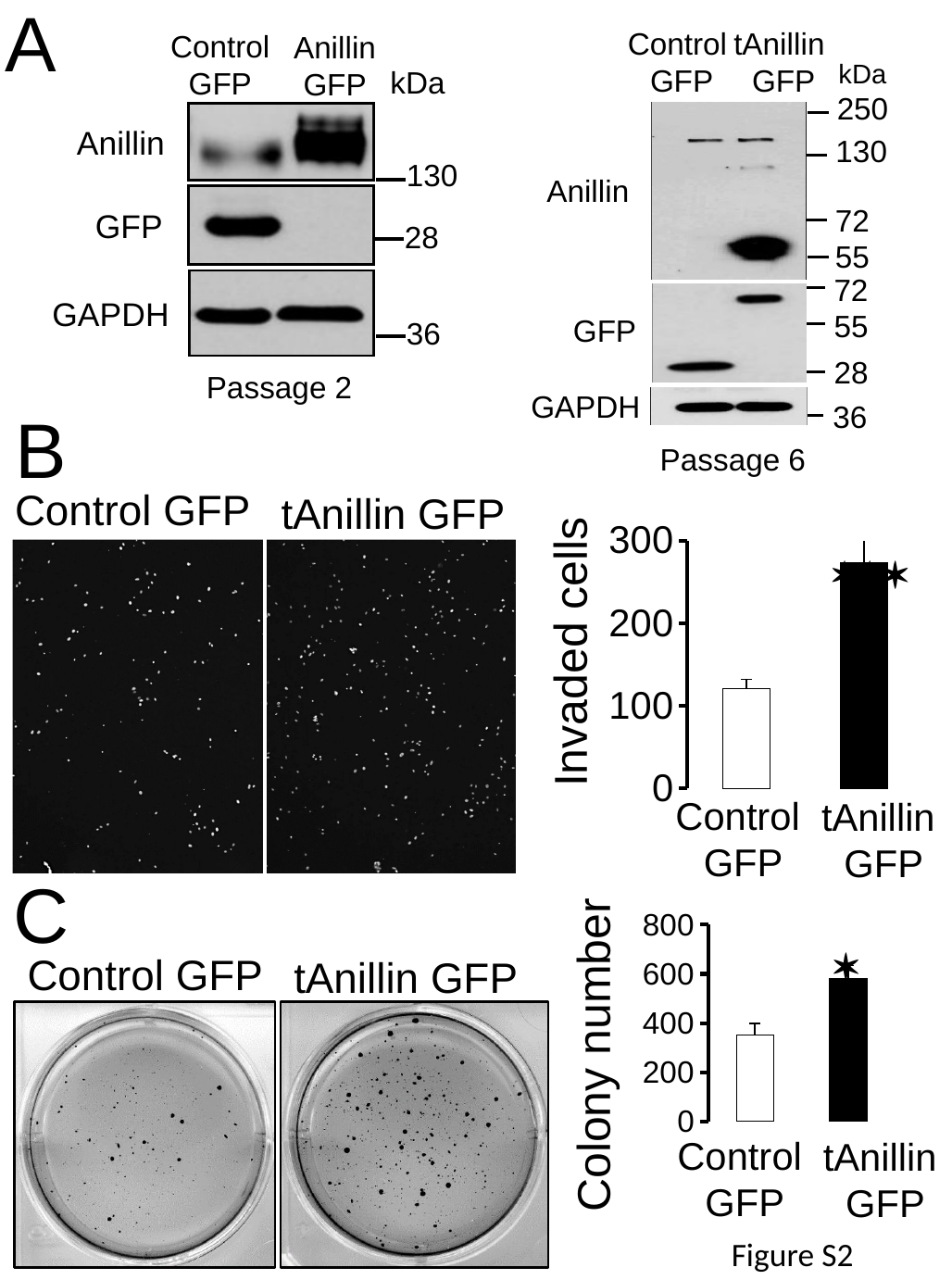

A
Control
GFP
tAnillin
 GFP
Control GFP
Anillin GFP
kDa
kDa
250
Anillin
130
130
Anillin
72
GFP
28
55
72
GAPDH
55
GFP
36
28
Passage 2
GAPDH
36
B
Passage 6
Control GFP
tAnillin GFP
### Chart
| Category | |
|---|---|
| Control GFP | 121.5 |
| Anillin GFP | 274.0 |
Invaded cells
Control
GFP
tAnillin
 GFP
C
### Chart
| Category | |
|---|---|
| Control GFP | 349.5 |
| Anillin GFP | 582.0 |
Control GFP
tAnillin GFP
Colony number
Control
GFP
tAnillin
 GFP
Figure S2
