# Supplementary figures and images for: Anillin regulates breast cancer cell migration, growth, and metastasis by non-canonical mechanisms involving control of cell stemness and differentiation
Source: Breast Cancer Res. 2020 Jan 7;22:3. doi: 10.1186/s13058-019-1241-x (PMC6947866; doi:10.1186/s13058-019-1241-x)

## Slide 1
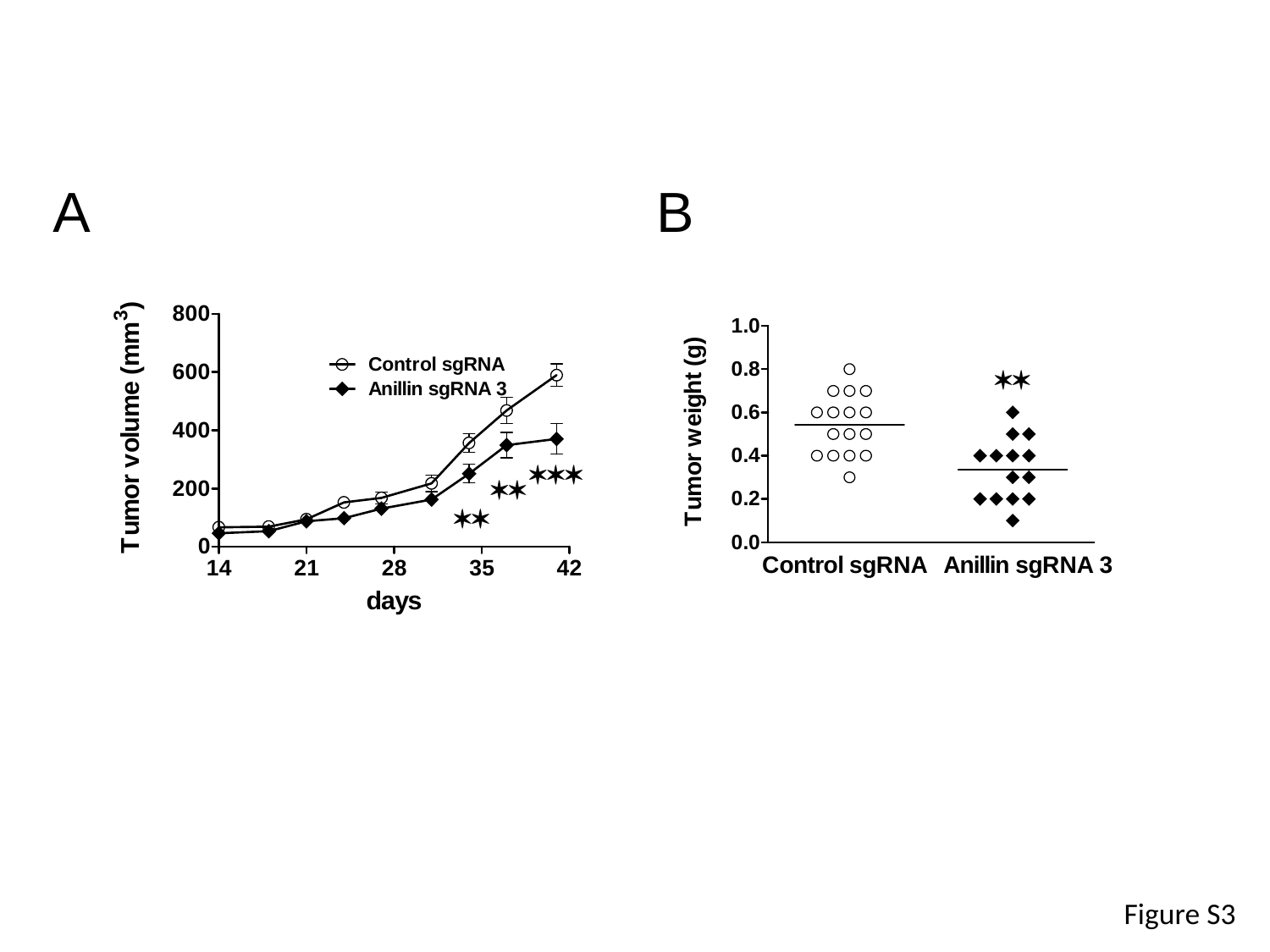

A
B




Figure S3

Supplement: Supplementary file 4 — Figure S3. Anillin depletion with sgRNA 3 inhibits growth of primary breast tumor in vivo. Control and anillin-sgRNA 3-depleted MDA-MB-231 cells were injected into the mammary gland of NSG mice. Tumor volume was measured starting at day 14 after injection of the cells (A), whereas weight of the dissected tumors was measured at the end of the experiment (B). Data is presented as mean ± SE (n = 12–14); **p < 0.01; ***p < 0.001. [file 13058_2019_1241_MOESM4_ESM.pptx]

## Slide 1
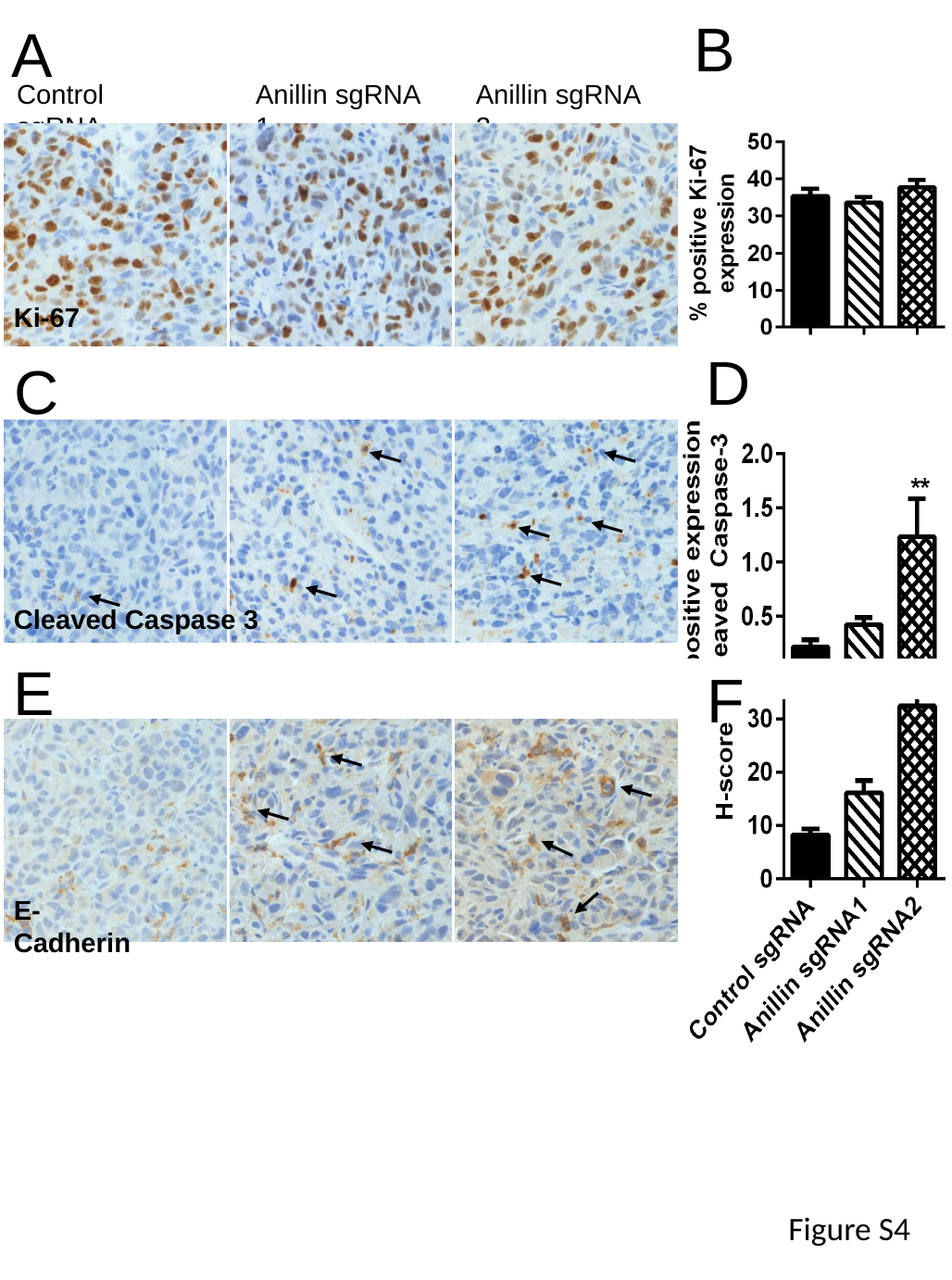

Anillin sgRNA 1
Anillin sgRNA 2
Control sgRNA
B
A
Ki-67
D
C
Cleaved Caspase 3
E
F
E-Cadherin
Figure S4

Supplement: Supplementary file 5 — Figure S4. Anillin depletion increases E-cadherin and cleaved caspase-3 expression in MDA-MB-231cell-derived breast tumors in vivo. Control and two different anillin-knockout MDA-MB-231 cell lines (Anillin sgRNA 1 and sgRNA 2) were injected into the mammary gland of NSG mice. Ki-67, cleaved caspase-3, and E-Cadherin levels were examined by IHC staining of the mammary tumors. The staining was imaged with Vectra Polaris (A,C,E) and quantified (B,D,F) using Inform software. Data are presented as mean ± SE (n = 4); **p < 0.001; ****p < 0.0001. Arrows, cleaved caspase-3 and E-cadherin staining. [file 13058_2019_1241_MOESM5_ESM.pptx]

## Slide 1
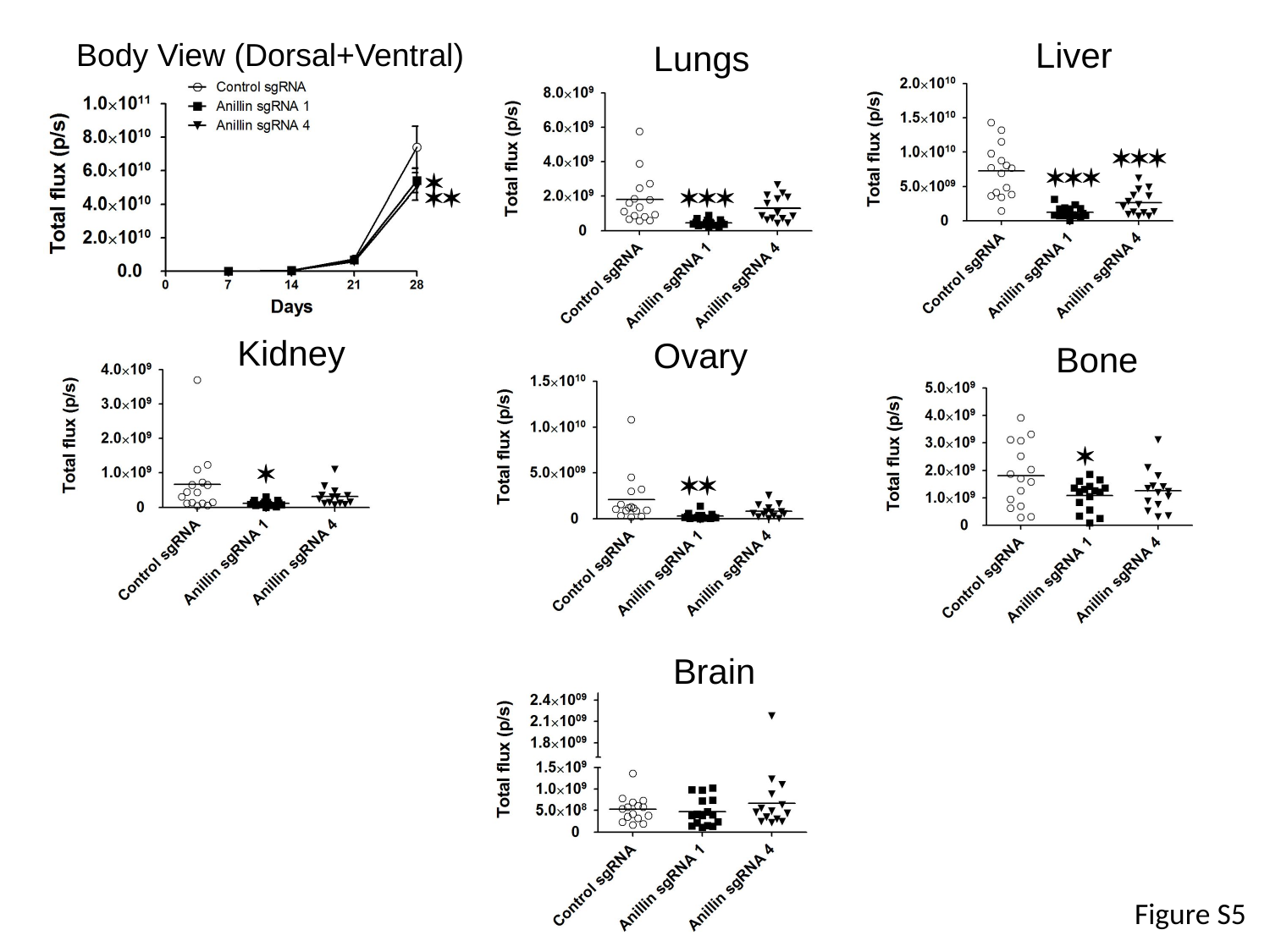

Liver
Body View (Dorsal+Ventral)
Lungs





Kidney
Ovary
Bone



Brain
Figure S5

Supplement: Supplementary file 6 — Figure S5. Loss of anillin inhibits breast cancer cell metastasis in vivo. Control and anillin sgRNA1 and sgRNA4-depleted MDA-MB-231 cell lines stable-expressing a luciferase construct were injected intracardially in NSG mice. Luciferase intensity of the dorsal and ventral side of the mouse was monitored starting on 7 days after injection. Four weeks after the injection, luciferase intensity of isolated lungs, liver, kidney, ovary, bones and brain was measured by IVIS. Data is presented as mean ± SE (n = 12–14); *p < 0.05; **p < 0.01; ***p < 0.001. [file 13058_2019_1241_MOESM6_ESM.pptx]

## Slide 1
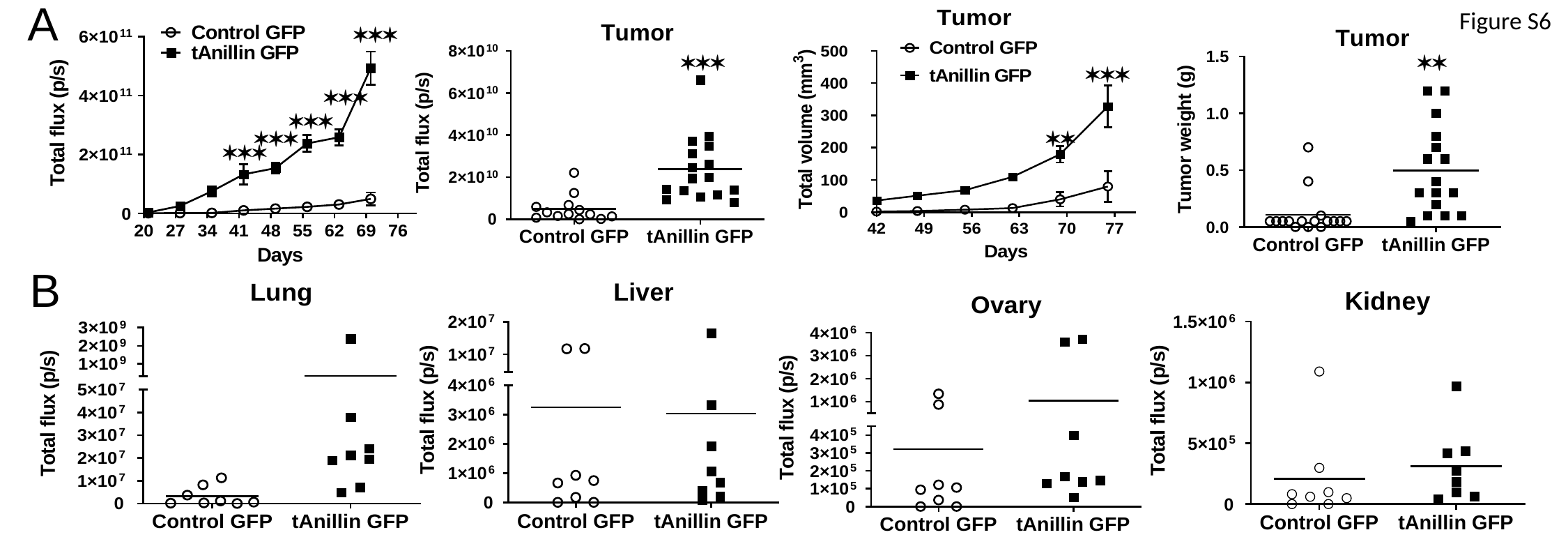

Figure S6
A









B

Supplement: Supplementary file 7 — Figure S6. Truncated anillin fragment stimulates primary breast tumor growth in vivo. MCF10AneoT cells stably expressing either truncated anillin-GFP (tAnillin) or control GFP along with a luciferase construct were injected into the mammary gland of NSG mice. (A) Luciferase intensity at the ventral side of the mouse and tumor volume were measured starting at days 20 and 42 after injection of the cells, respectively, whereas volume, weight and total luciferase intensity of dissected tumors was measured ten weeks after the injection at the end of the experiment. (B) Luciferase intensity in isolated lungs, liver, ovary, and kidney, was measured by IVIS at the end of the experiment. Data is presented as mean ± SE (n = 10–11); **p < 0.01; ***p < 0.001. [file 13058_2019_1241_MOESM7_ESM.pptx]

## Slide 1
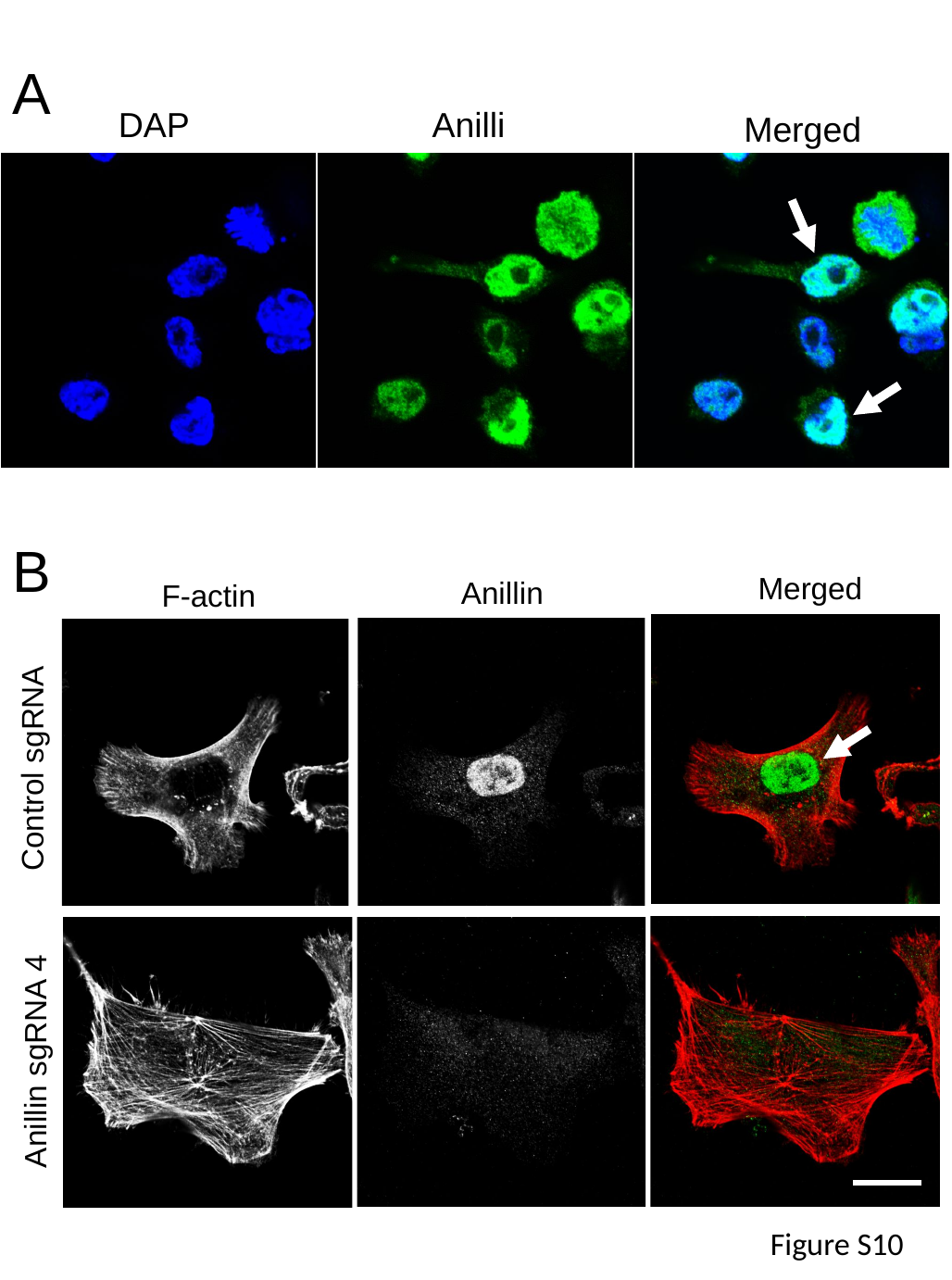

A
Anillin
DAPI
Merged
B
Merged
Anillin
F-actin
Control sgRNA
Anillin sgRNA 4
Figure S10

Supplement: Supplementary file 11 — Figure S10. Nuclear localization of anillin is the in invasive breast cancer cells. (A) Parental MDA-MB-231 cell were immunofluorescence labeled for anillin (green), whereas DAPI (blue) was used to label nuclei. (B) Control and anillin-depleted MDA-MB-231 cells were dual-immunolabeled for anillin (green) and F-actin (red). Arrow point on nuclear localization of anillin that disappeared in anillin-deficient cells. Scale bar, 20 μm. [file 13058_2019_1241_MOESM11_ESM.pptx]
