# Supplementary material for: Anillin regulates breast cancer cell migration, growth, and metastasis by non-canonical mechanisms involving control of cell stemness and differentiation
Source: Breast Cancer Res. 2020 Jan 7;22:3. doi: 10.1186/s13058-019-1241-x (PMC6947866; doi:10.1186/s13058-019-1241-x)
Supplement: Supplementary file 8 — Figure S7. Anillin depletion does not affect expression and activation of ECM adhesion proteins. Immunoblotting analysis of focal adhesion proteins and integrin subunits expression in control and anillin-depleted MDA-MB-231 cells. [file 13058_2019_1241_MOESM8_ESM.pptx]

## Slide 1
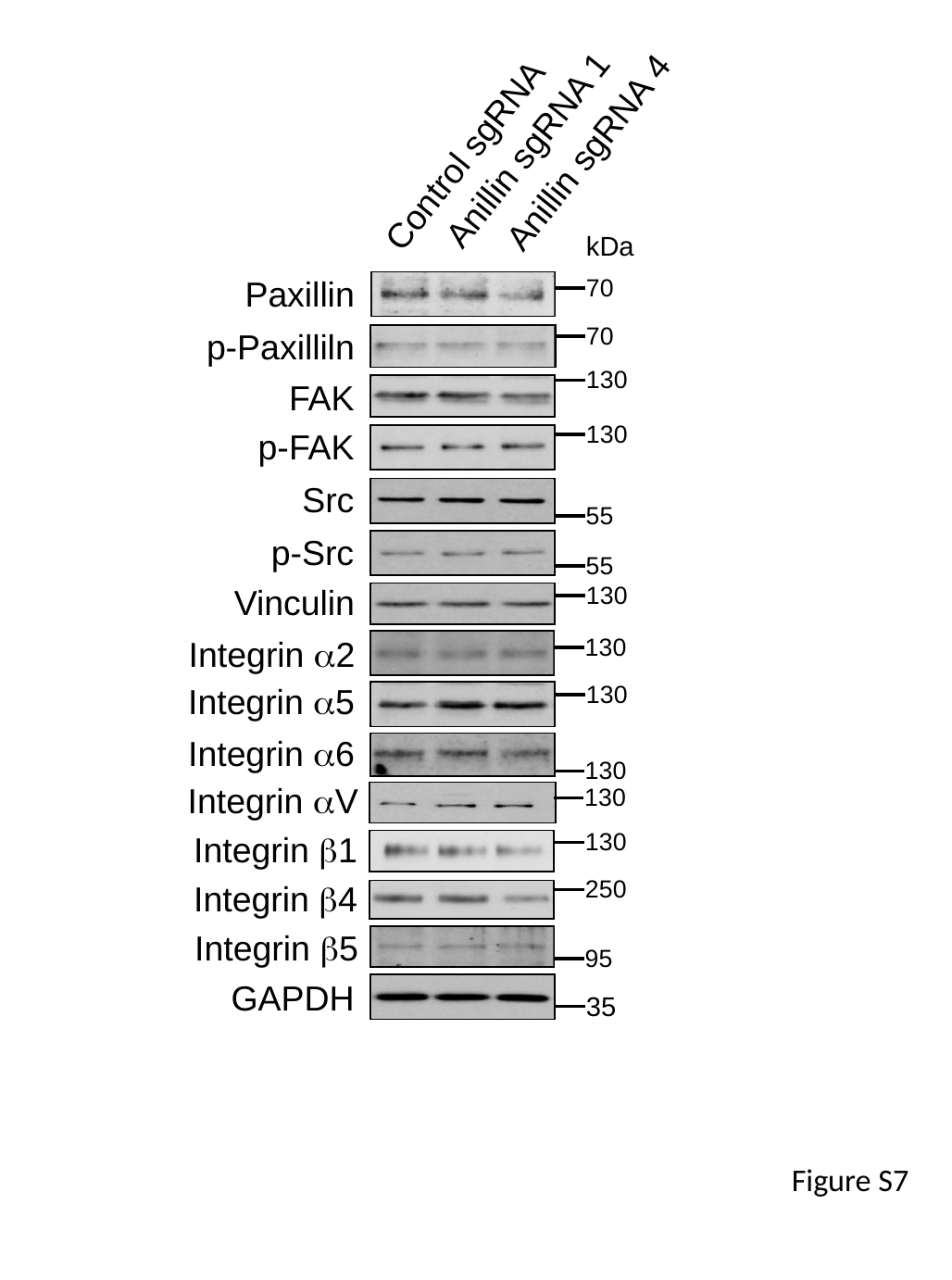

Anillin sgRNA 1
Anillin sgRNA 4
Control sgRNA
kDa
70
Paxillin
70
p-Paxilliln
130
FAK
130
p-FAK
Src
55
p-Src
55
130
Vinculin
130
Integrin α2
130
Integrin α5
Integrin α6
130
Integrin αV
130
130
Integrin β1
250
Integrin β4
Integrin β5
95
GAPDH
35
Figure S7
