# Supplementary material for: Anillin regulates breast cancer cell migration, growth, and metastasis by non-canonical mechanisms involving control of cell stemness and differentiation
Source: Breast Cancer Res. 2020 Jan 7;22:3. doi: 10.1186/s13058-019-1241-x (PMC6947866; doi:10.1186/s13058-019-1241-x)
Supplement: Supplementary file 9 — Figure S8. Inhibition of NM II reverses attenuated collective migration of anillin-depleted cells. Control and anillin-knockout MDA-MB-231 cells were incubated with either vehicle, or a NM II inhibitor, blebbistatin (50 μM). (A) The effect of blebbistatin on the actin cytoskeleton architecture was determined by phalloidin labeling and confocal microscopy. Arrow indicates stress fibers in vehicle treated anillin-depleted cells, whereas arrowhead points on disappearance of stress fibers in blebbistatin-exposed anillin-depleted cells. (B) The effects of blebbistain on collective cell migration was examined during 12 h wound closure assay. Data is presented as mean ± SE (n = 3); **p < 0.01. Scale bar, 20 μm. [file 13058_2019_1241_MOESM9_ESM.pptx]

## Slide 1
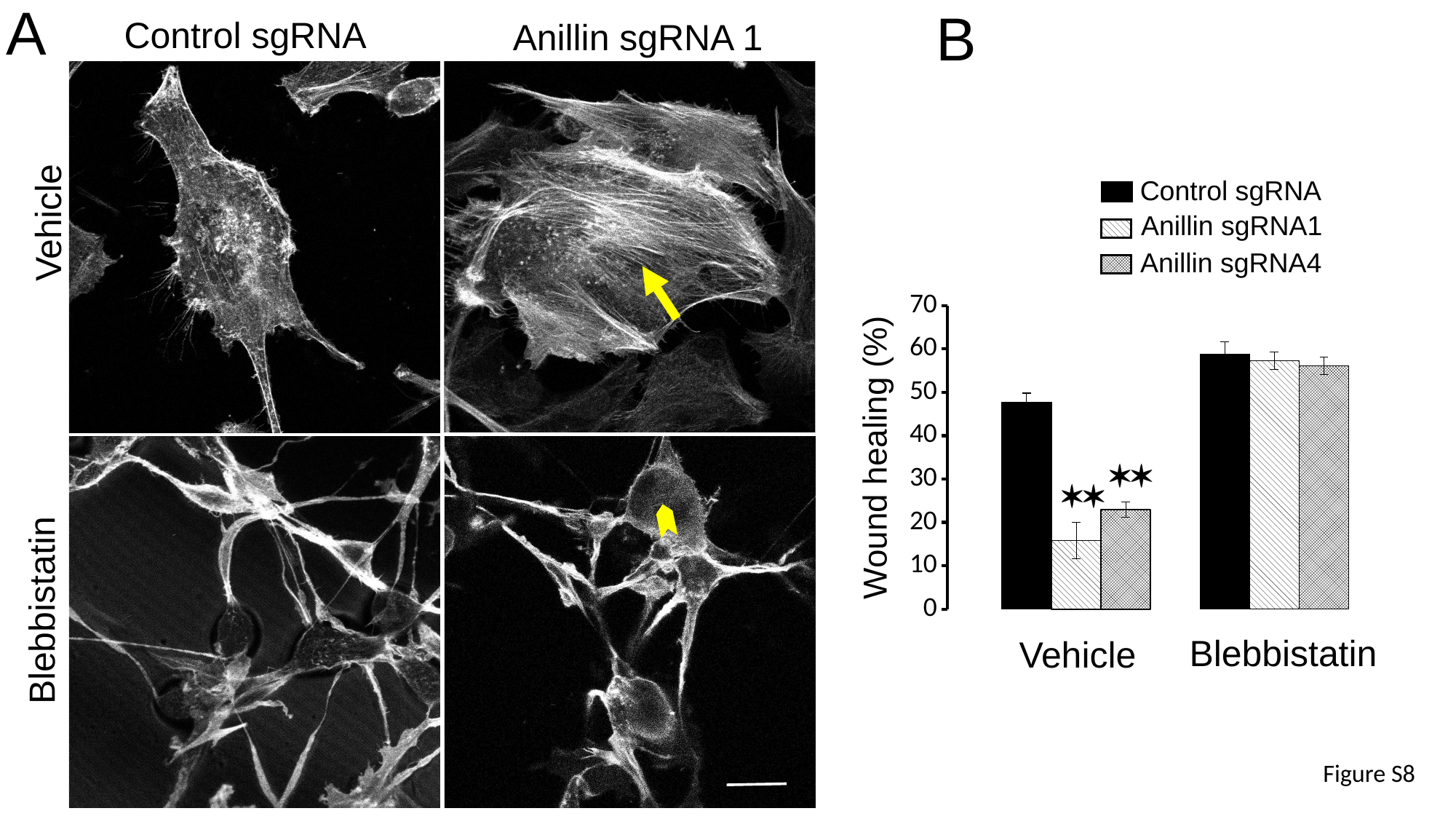

A
B
Control sgRNA
Anillin sgRNA 1
Control sgRNA
Vehicle
Anillin sgRNA1
Anillin sgRNA4
### Chart
| Category | | | | | | | |
|---|---|---|---|---|---|---|---|Wound healing (%)
Blebbistatin
Blebbistatin
Vehicle
Figure S8
