# Supplementary material for: Anillin regulates breast cancer cell migration, growth, and metastasis by non-canonical mechanisms involving control of cell stemness and differentiation
Source: Breast Cancer Res. 2020 Jan 7;22:3. doi: 10.1186/s13058-019-1241-x (PMC6947866; doi:10.1186/s13058-019-1241-x)
Supplement: Supplementary file 10 — Figure S9. Anillin depletion does not alter the activity of RhoA and Rac1 small GTPases. The amount of active RhoA in control and anillin-depleted MDA-MB-231 cells was determined by a specific G-LISA assay (A), whereas Rac1 activity was examined by a pull-down of active Rac1 with subsequent immunoblotting analysis of active and total Rac1 expression (B). [file 13058_2019_1241_MOESM10_ESM.pptx]

## Slide 1
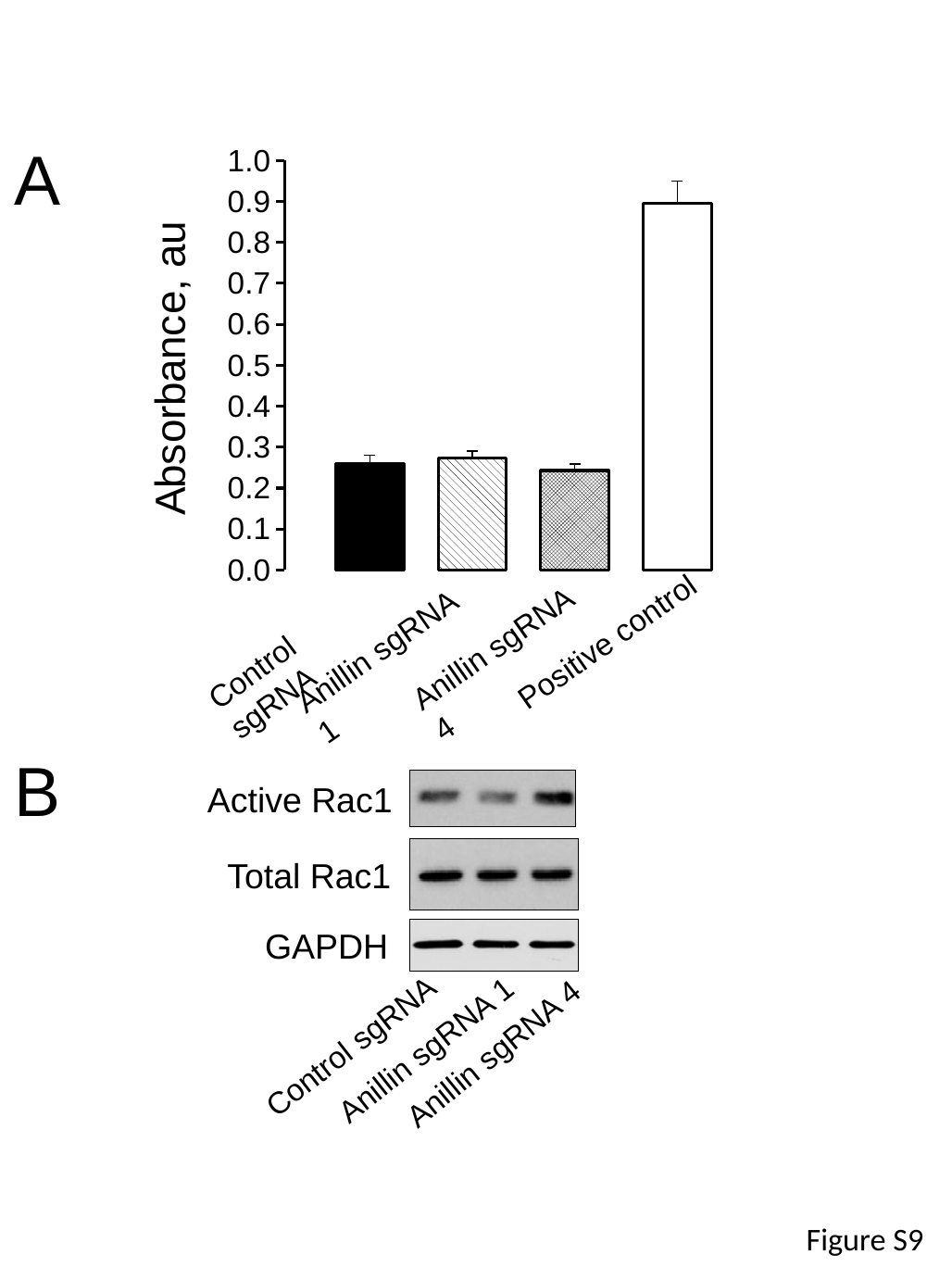

### Chart
| Category | Control sgRNA 2 | Anillin sgRNA 1 | Anillin sgRNA 4 | Positive control |
|---|---|---|---|---|
| 490 nm 0.1 S | 0.2596986751599907 | 0.27361677193107853 | 0.24264098640848836 | 0.8961483421581787 |A
Positive control
Control sgRNA
Anillin sgRNA 1
Anillin sgRNA 4
B
Active Rac1
Total Rac1
GAPDH
Control sgRNA
Anillin sgRNA 1
Anillin sgRNA 4
Figure S9
