# Supplementary material for: Anillin regulates breast cancer cell migration, growth, and metastasis by non-canonical mechanisms involving control of cell stemness and differentiation
Source: Breast Cancer Res. 2020 Jan 7;22:3. doi: 10.1186/s13058-019-1241-x (PMC6947866; doi:10.1186/s13058-019-1241-x)
Supplement: Supplementary file 12 — Table S2. List of genes upregulated in anillin-deficient MDA-MB-231 cell lines as compared to control cells. “Geneid”, “symbol”, “description” - gene annotations; “logFC.1”, “logFC.2” - log fold change in the first and second CRISPR experiments, respectively; “Average logFC” – average log fold change; “FDR.1”, “FDR.2” - FDR-adjusted p-value of differential expression in the first and second CRISPR experiments, respectively. [file 13058_2019_1241_MOESM12_ESM.pdf]

Supplementary Table S2. List of genes upregulated in anillin-deficient MDA-MB-231 cell lines as compared to control cells.

"Geneid", "symbol", "description" - gene annotations; "logFC.1", "logFC.2" - log fold change in the first and second CRISPR experiments, respectively;
Average logFC" – average log fold change; "FDR.1", "FDR.2" - FDR-adjusted p-value of differential expression in the first and second CRISPR experiments, respectively.

Geneid	logFC.1	FDR.1	logFC.2	FDR.2	Average logFC	symbol	description
ENSG0000	5.191235	1.05E-22	6.805635	1.51E-39	6.00	KRT5	keratin 5, type II [Source:HGNC Symbol;Acc:HGNC:6442]
ENSG0000	3.177175	0.000118	4.425871	9.31E-14	3.80	KRT14	keratin 14, type I [Source:HGNC Symbol;Acc:HGNC:6416]
ENSG0000	2.59171	0.010452	4.286461	1.06E-11	3.44	FXYD3	FXYD domain containing ion transport regulator 3 [Source:HGNC Symbol;Acc:HGNC:4027]
ENSG0000	3.572329	0.009345	2.950018	0.03658	3.26	ID2	inhibitor of DNA binding 2, dominant negative helix-loop-helix protein [Source:HGNC Symbol;Acc:HGNC:5361]
ENSG0000	1.561186	0.010945	4.870102	2.41E-47	3.22	KRT6A	keratin 6A, type II [Source:HGNC Symbol;Acc:HGNC:6443]
ENSG0000	2.26426	6.21E-07	2.390247	5.99E-09	2.33	S100A14	S100 calcium binding protein A14 [Source:HGNC Symbol;Acc:HGNC:18901]
ENSG0000	1.966062	0.030373	2.466117	0.00247	2.22	KRT6C	keratin 6C, type II [Source:HGNC Symbol;Acc:HGNC:20406]
ENSG0000	2.535002	2.03E-13	1.721424	2.11E-05	2.13	TRNP1	TMF1-regulated nuclear protein 1 [Source:HGNC Symbol;Acc:HGNC:34348]
ENSG0000	2.29446	0.002283	1.744097	0.01837	2.02	SNHG19	small nucleolar RNA host gene 19 [Source:HGNC Symbol;Acc:HGNC:49574]
ENSG0000	2.213019	0.005173	1.817592	0.019618	2.02	RPS24P8	ribosomal protein S24 pseudogene 8 [Source:HGNC Symbol;Acc:HGNC:37016]
ENSG0000	1.159561	0.023739	2.676866	4.11E-14	1.92	KRT6B	keratin 6B, type II [Source:HGNC Symbol;Acc:HGNC:6444]
ENSG0000	2.444137	7.31E-13	1.34619	0.002892	1.90	CA2	carbonic anhydrase II [Source:HGNC Symbol;Acc:HGNC:1373]
ENSG0000	2.18412	0.004091	1.567089	0.033215	1.88	ATP5I	ATP synthase, H+ transporting, mitochondrial Fo complex, subunit E [Source:HGNC Symbol;Acc:HGNC:846]
ENSG0000	2.053339	1.97E-06	1.585704	0.000807	1.82	DMKN	dermokine [Source:HGNC Symbol;Acc:HGNC:25063]
ENSG0000	1.415831	0.002514	2.121103	4E-08	1.77	KRT17	keratin 17, type I [Source:HGNC Symbol;Acc:HGNC:6427]
ENSG0000	1.893473	0.006417	1.595668	0.019994	1.74	CASP1	caspase 1, apoptosis-related cysteine peptidase [Source:HGNC Symbol;Acc:HGNC:1499]
ENSG0000	2.159028	0.000762	1.329308	0.034252	1.74	RPL41	ribosomal protein L41 [Source:HGNC Symbol;Acc:HGNC:10354]
ENSG0000	2.025157	0.006175	1.425193	0.04935	1.73	SLIRP	SRA stem-loop interacting RNA binding protein [Source:HGNC Symbol;Acc:HGNC:20495]
ENSG0000	2.145586	3.63E-06	1.30208	0.013482	1.72	S100P	S100 calcium binding protein P [Source:HGNC Symbol;Acc:HGNC:10504]
ENSG0000	1.994439	0.005819	1.435636	0.041144	1.72	RPS21	ribosomal protein S21 [Source:HGNC Symbol;Acc:HGNC:10409]
ENSG0000	2.021472	0.002448	1.302798	0.046794	1.66	LINC00493	long intergenic non-protein coding RNA 493 [Source:HGNC Symbol;Acc:HGNC:43430]
ENSG0000	1.882375	0.000325	1.40877	0.009575	1.65	TAGLN	transgelin [Source:HGNC Symbol;Acc:HGNC:11553]
ENSG0000	2.138867	1.32E-05	1.120827	0.034252	1.63	PCBD1	pterin-4 alpha-carbinolamine dehydratase/dimerization cofactor of hepatocyte nuclear factor 1 alpha [Source:HGNC Symbol;Acc:HGNC:8646]
ENSG0000	1.981834	0.002864	1.277759	0.04801	1.63	COX7C	cytochrome c oxidase subunit VIIc [Source:HGNC Symbol;Acc:HGNC:2292]
ENSG0000	1.595848	0.026486	1.641244	0.021574	1.62	SAA1	serum amyloid A1 [Source:HGNC Symbol;Acc:HGNC:10513]
ENSG0000	1.878339	0.002149	1.341982	0.024999	1.61	RPL22L1	ribosomal protein L22-like 1 [Source:HGNC Symbol;Acc:HGNC:27610]
ENSG0000	1.940176	0.002514	1.267095	0.041607	1.60	RPL31	ribosomal protein L31 [Source:HGNC Symbol;Acc:HGNC:10334]
ENSG0000	1.955929	5.97E-05	1.225489	0.016278	1.59	SNRPF	small nuclear ribonucleoprotein polypeptide F [Source:HGNC Symbol;Acc:HGNC:11162]
ENSG0000	1.918648	0.001401	1.255811	0.036041	1.59	SNRPFP1	small nuclear ribonucleoprotein polypeptide F pseudogene 1 [Source:HGNC Symbol;Acc:HGNC:16551]
ENSG0000	1.8757	0.005073	1.295325	0.04758	1.59	COX6B1	cytochrome c oxidase subunit VIb polypeptide 1 (ubiquitous) [Source:HGNC Symbol;Acc:HGNC:2280]
ENSG0000	1.868254	0.001155	1.30131	0.020996	1.58	COX6C	cytochrome c oxidase subunit VIc [Source:HGNC Symbol;Acc:HGNC:2285]
ENSG0000	1.801228	0.005882	1.329268	0.036875	1.57	HSPE1	heat shock 10kDa protein 1 [Source:HGNC Symbol;Acc:HGNC:5269]
ENSG0000	1.654521	0.0144	1.472255	0.027298	1.56	SEL1L3	sel-1 suppressor of lin-12-like 3 (C. elegans) [Source:HGNC Symbol;Acc:HGNC:29108]
ENSG0000	1.883555	0.00259	1.223868	0.045348	1.55	TMEM258	transmembrane protein 258 [Source:HGNC Symbol;Acc:HGNC:1164]
ENSG0000	1.821312	0.005357	1.268214	0.047805	1.54	RPL35P5	ribosomal protein L35 pseudogene 5 [Source:HGNC Symbol;Acc:HGNC:36955]
ENSG0000	1.793079	0.006842	1.295094	0.047939	1.54	UQCR10	ubiquinol-cytochrome c reductase, complex III subunit X [Source:HGNC Symbol;Acc:HGNC:30863]
ENSG0000	1.665973	0.021635	1.416877	0.048637	1.54	SNRPGP15	small nuclear ribonucleoprotein polypeptide G pseudogene 15 [Source:HGNC Symbol;Acc:HGNC:49371]
ENSG0000	1.860862	0.001519	1.221191	0.032762	1.54	DBI	diazepam binding inhibitor (GABA receptor modulator, acyl-CoA binding protein) [Source:HGNC Symbol;Acc:HGNC:2690]
ENSG0000	1.816695	0.004963	1.252127	0.04711	1.53	COX5B	cytochrome c oxidase subunit Vb [Source:HGNC Symbol;Acc:HGNC:2269]
ENSG0000	1.867941	0.002432	1.191148	0.046794	1.53	RPL36AL	ribosomal protein L36a-like [Source:HGNC Symbol;Acc:HGNC:10346]
ENSG0000	1.785438	0.006224	1.270592	0.048637	1.53	DNPH1	2'-deoxynucleoside 5'-phosphate N-hydrolase 1 [Source:HGNC Symbol;Acc:HGNC:21218]
ENSG0000	1.840237	0.000827	1.180208	0.029547	1.51	COX6A1P2	cytochrome c oxidase subunit VIa polypeptide 1 pseudogene 2 [Source:HGNC Symbol;Acc:HGNC:35239]
ENSG0000	1.773159	0.001413	1.235211	0.022311	1.50	UQCRB	ubiquinol-cytochrome c reductase binding protein [Source:HGNC Symbol;Acc:HGNC:12582]
ENSG0000	1.920687	8.09E-06	1.059073	0.029208	1.49	EMB	embigin [Source:HGNC Symbol;Acc:HGNC:30465]
ENSG0000	1.709357	0.004747	1.269203	0.031037	1.49	BOLA3	bolA family member 3 [Source:HGNC Symbol;Acc:HGNC:24415]
ENSG0000	1.800425	1.41E-05	1.165607	0.010259	1.48	FABP5P7	fatty acid binding protein 5 pseudogene 7 [Source:HGNC Symbol;Acc:HGNC:31070]
ENSG0000	1.784654	0.000108	1.170793	0.015678	1.48	FAM216A	family with sequence similarity 216, member A [Source:HGNC Symbol;Acc:HGNC:30180]
ENSG0000	1.783415	0.000572	1.136695	0.027384	1.46	NDUFA12	NADH dehydrogenase (ubiquinone) 1 alpha subcomplex, 12 [Source:HGNC Symbol;Acc:HGNC:23987]
ENSG0000	1.774055	0.000952	1.134386	0.034108	1.45	POP5	POP5 homolog, ribonuclease P/MRP subunit [Source:HGNC Symbol;Acc:HGNC:17689]
ENSG0000	1.687112	0.004747	1.155621	0.046703	1.42	RPL35	ribosomal protein L35 [Source:HGNC Symbol;Acc:HGNC:10344]
ENSG0000	1.620425	0.002714	1.210912	0.022311	1.42	HSPE1P4	heat shock 10kDa protein 1 pseudogene 4 [Source:HGNC Symbol;Acc:HGNC:49323]
ENSG0000	1.582981	0.008785	1.202943	0.044592	1.39	COX7A2	cytochrome c oxidase subunit VIIa polypeptide 2 (liver) [Source:HGNC Symbol;Acc:HGNC:2288]
ENSG0000	1.677667	0.0015	1.100892	0.034107	1.39	TXNP5	thioredoxin pseudogene 5 [Source:HGNC Symbol;Acc:HGNC:49485]
ENSG0000	1.599153	0.004855	1.13784	0.039018	1.37	TXN	thioredoxin [Source:HGNC Symbol;Acc:HGNC:12435]
ENSG0000	1.539107	0.006638	1.112708	0.047351	1.33	SNRPG	small nuclear ribonucleoprotein polypeptide G [Source:HGNC Symbol;Acc:HGNC:11163]
ENSG0000	1.606423	0.000462	1.028528	0.026461	1.32	ABRACL	ABRA C-terminal like [Source:HGNC Symbol;Acc:HGNC:21230]
ENSG0000	1.574855	0.001519	1.05866	0.029543	1.32	USMG5	up-regulated during skeletal muscle growth 5 homolog (mouse) [Source:HGNC Symbol;Acc:HGNC:30889]
ENSG0000	1.642508	3.71E-06	0.974495	0.013154	1.31	C12orf75	chromosome 12 open reading frame 75 [Source:HGNC Symbol;Acc:HGNC:35164]
ENSG0000	1.628964	0.000588	0.938244	0.048107	1.28	RPS26P6	ribosomal protein S26 pseudogene 6 [Source:HGNC Symbol;Acc:HGNC:31090]
ENSG0000	1.515777	0.000952	1.011512	0.025845	1.26	POLR2K	polymerase (RNA) II (DNA directed) polypeptide K, 7.0kDa [Source:HGNC Symbol;Acc:HGNC:9198]
ENSG0000	1.540155	0.000377	0.953996	0.028695	1.25	ARPC3	actin related protein 2/3 complex, subunit 3, 21kDa [Source:HGNC Symbol;Acc:HGNC:706]
ENSG0000	1.505931	0.001916	0.953258	0.044149	1.23	ENY2	enhancer of yellow 2 homolog (Drosophila) [Source:HGNC Symbol;Acc:HGNC:24449]
ENSG0000	1.408038	0.002109	0.887728	0.048958	1.15	TXNP6	thioredoxin pseudogene 6 [Source:HGNC Symbol;Acc:HGNC:49486]
ENSG0000	1.523556	2.29E-06	0.735757	0.041329	1.13	IL18	interleukin 18 [Source:HGNC Symbol;Acc:HGNC:5986]
ENSG0000	1.367318	0.000108	0.759264	0.035726	1.06	VPS29	VPS29 retromer complex component [Source:HGNC Symbol;Acc:HGNC:14340]
ENSG0000	1.210291	0.009356	0.910649	0.048637	1.06	TPD52L1	tumor protein D52-like 1 [Source:HGNC Symbol;Acc:HGNC:12006]
