# Supplementary material for: Anillin regulates breast cancer cell migration, growth, and metastasis by non-canonical mechanisms involving control of cell stemness and differentiation
Source: Breast Cancer Res. 2020 Jan 7;22:3. doi: 10.1186/s13058-019-1241-x (PMC6947866; doi:10.1186/s13058-019-1241-x)
Supplement: Supplementary file 13 — Table S3. List of genes downregulated in anillin-deficient MDA-MB-231 cell lines as compared to control cells. “Geneid”, “symbol”, “description” - gene annotations; “logFC.1”, “logFC.2” - log fold change in the first and second CRISPR experiments, respectively; “Average logFC” – average log fold change; “FDR.1”, “FDR.2” - FDR-adjusted p-value of differential expression in the first and second CRISPR experiments, respectively. [file 13058_2019_1241_MOESM13_ESM.pdf]

Supplementary Table S3. List of genes downregulated in anillin-deficient MDA-MB-231 cell lines as compared to control cells.
"Geneid", "symbol", "description" - gene annotations; "logFC.1", "logFC.2" - log fold change in the first and second CRISPR experiments, respectively;
Average logFC" – average log fold change; "FDR.1", "FDR.2" - FDR-adjusted p-value of differential expression in the first and second CRISPR experiments, respectively.

Geneid	logFC.1	FDR.1	logFC.2	FDR.2	Average logFC	symbol	description
ENSG000(	-3.6301	0.00622	-4.9385	0.0006	-4.28	IGSF9B	immunoglobulin superfamily, member 9B [Source:HGNC Symbol;Acc:HGNC:32326]
ENSG000(	-3.6267	0.02559	-3.9362	0.01165	-3.78	MARVELD	MARVEL domain containing 3 [Source:HGNC Symbol;Acc:HGNC:30525]
ENSG000(	-3.5635	0.04101	-3.8574	0.01838	-3.71	TBX18	T-box 18 [Source:HGNC Symbol;Acc:HGNC:11595]
ENSG000(	-3.6136	0.00787	-3.1607	0.00713	-3.39	ANO1	anoctamin 1, calcium activated chloride channel [Source:HGNC Symbol;Acc:HGNC:21625]
ENSG000(	-3.1557	0.01079	-2.6389	0.01313	-2.90	GATM	glycine amidinotransferase (L-arginine:glycine amidinotransferase) [Source:HGNC Symbol;Acc:HGNC:4175]
ENSG000(	-3.2224	0.02	-2.5503	0.02584	-2.89	OVOL2	ovo-like zinc finger 2 [Source:HGNC Symbol;Acc:HGNC:15804]
ENSG000(	-2.3667	0.00635	-2.6471	0.00144	-2.51	BMP2	bone morphogenetic protein 2 [Source:HGNC Symbol;Acc:HGNC:1069]
ENSG000(	-2.1329	0.02012	-2.5261	0.00636	-2.33	SORL1	sortilin-related receptor, L(DLR class) A repeats containing [Source:HGNC Symbol;Acc:HGNC:11185]
ENSG000(	-2.2082	0.03732	-2.4127	0.01601	-2.31	SLC6A11	solute carrier family 6 (neurotransmitter transporter), member 11 [Source:HGNC Symbol;Acc:HGNC:11044]
ENSG000(	-2.325	4.2E-11	-2.2343	1.1E-11	-2.28	PPL	periplakin [Source:HGNC Symbol;Acc:HGNC:9273]
ENSG000(	-2.4332	0.01321	-2.0809	0.0232	-2.26	ADAM12	ADAM metallopeptidase domain 12 [Source:HGNC Symbol;Acc:HGNC:190]
ENSG000(	-2.1692	0.01803	-2.3337	0.01255	-2.25	HIPK2	homeodomain interacting protein kinase 2 [Source:HGNC Symbol;Acc:HGNC:14402]
ENSG000(	-2.7247	0.00025	-1.5584	0.01823	-2.14	TFCP2L1	transcription factor CP2-like 1 [Source:HGNC Symbol;Acc:HGNC:17925]
ENSG000(	-2.0502	0.03619	-2.2028	0.02231	-2.13	FOXK1	forkhead box K1 [Source:HGNC Symbol;Acc:HGNC:23480]
ENSG000(	-1.7101	0.04662	-2.371	0.00705	-2.04	MBNL3	muscleblind-like splicing regulator 3 [Source:HGNC Symbol;Acc:HGNC:20564]
ENSG000(	-2.0404	0.04003	-2.0365	0.0267	-2.04	PBX1	pre-B-cell leukemia homeobox 1 [Source:HGNC Symbol;Acc:HGNC:8632]
ENSG000(	-2.1972	0.01045	-1.5994	0.02778	-1.90	SCEL	sciellin [Source:HGNC Symbol;Acc:HGNC:10573]
ENSG000(	-1.692	0.0209	-2.012	0.00797	-1.85	AHNAK	AHNAK nucleoprotein [Source:HGNC Symbol;Acc:HGNC:347]
ENSG000(	-1.7105	0.02374	-1.9494	0.01157	-1.83	FAT1	FAT atypical cadherin 1 [Source:HGNC Symbol;Acc:HGNC:3595]
ENSG000(	-1.7176	0.0069	-1.9411	0.00251	-1.83	AHR	aryl hydrocarbon receptor [Source:HGNC Symbol;Acc:HGNC:348]
ENSG000(	-1.6484	0.02646	-1.944	0.01019	-1.80	AKAP9	A kinase (PRKA) anchor protein 9 [Source:HGNC Symbol;Acc:HGNC:379]
ENSG000(	-1.7886	0.00691	-1.7948	0.00719	-1.79	USP9X	ubiquitin specific peptidase 9, X-linked [Source:HGNC Symbol;Acc:HGNC:12632]
ENSG000(	-1.5627	0.03854	-1.9326	0.01136	-1.75	AHNAK2	AHNAK nucleoprotein 2 [Source:HGNC Symbol;Acc:HGNC:20125]
ENSG000(	-1.7056	0.00963	-1.774	0.00792	-1.74	ATRX	alpha thalassemia/mental retardation syndrome X-linked [Source:HGNC Symbol;Acc:HGNC:886]
ENSG000(	-1.7068	0.00076	-1.6459	0.00108	-1.68	DSP	desmoplakin [Source:HGNC Symbol;Acc:HGNC:3052]
ENSG000(	-1.5049	0.03699	-1.8113	0.01175	-1.66	SLC9A7	solute carrier family 9, subfamily A (NHE7, cation proton antiporter 7), member 7 [Source:HGNC Symbol;Acc:HGNC:17123]
ENSG000(	-1.4662	0.0451	-1.7944	0.01314	-1.63	KMT2E	lysine (K)-specific methyltransferase 2E [Source:HGNC Symbol;Acc:HGNC:18541]
ENSG000(	-1.5568	0.01478	-1.6951	0.00817	-1.63	CHD7	chromodomain helicase DNA binding protein 7 [Source:HGNC Symbol;Acc:HGNC:20626]
ENSG000(	-1.6014	0.01739	-1.6448	0.01348	-1.62	PLEKHA2	pleckstrin homology domain containing, family A (phosphoinositide binding specific) member 2 [Source:HGNC Symbol;Acc:HGNC:14336]
ENSG000(	-1.4839	0.02	-1.7216	0.00792	-1.60	ARHGAP3	Rho GTPase activating protein 32 [Source:HGNC Symbol;Acc:HGNC:17399]
ENSG000(	-1.6402	0.02574	-1.5237	0.03645	-1.58	MALAT1	metastasis associated lung adenocarcinoma transcript 1 (non-protein coding) [Source:HGNC Symbol;Acc:HGNC:29665]
ENSG000(	-1.5737	0.03153	-1.5816	0.02629	-1.58	XIAP	X-linked inhibitor of apoptosis, E3 ubiquitin protein ligase [Source:HGNC Symbol;Acc:HGNC:592]
ENSG000(	-1.409	0.03682	-1.7268	0.01111	-1.57	RNF213	ring finger protein 213 [Source:HGNC Symbol;Acc:HGNC:14539]
ENSG000(	-1.4465	0.04003	-1.6887	0.01608	-1.57	HCFC1	host cell factor C1 [Source:HGNC Symbol;Acc:HGNC:4839]
ENSG000(	-1.4453	0.03207	-1.6816	0.01293	-1.56	TRRAP	transformation/transcription domain-associated protein [Source:HGNC Symbol;Acc:HGNC:12347]
ENSG000(	-1.3623	0.00531	-1.7633	0.00014	-1.56	EHF	ets homologous factor [Source:HGNC Symbol;Acc:HGNC:3246]
ENSG000(	-1.4729	0.03106	-1.6517	0.01632	-1.56	SPTBN1	spectrin, beta, non-erythrocytic 1 [Source:HGNC Symbol;Acc:HGNC:11275]
ENSG000(	-1.5483	0.02704	-1.573	0.025	-1.56	THBS1	thrombospondin 1 [Source:HGNC Symbol;Acc:HGNC:11785]
ENSG000(	-1.3488	0.04636	-1.7654	0.00976	-1.56	MACF1	microtubule-actin crosslinking factor 1 [Source:HGNC Symbol;Acc:HGNC:13664]
ENSG000(	-1.4501	0.03614	-1.6275	0.01823	-1.54	DDX6	DEAD (Asp-Glu-Ala-Asp) box helicase 6 [Source:HGNC Symbol;Acc:HGNC:2747]
ENSG000(	-1.4261	0.03929	-1.6484	0.01658	-1.54	NAV2	neuron navigator 2 [Source:HGNC Symbol;Acc:HGNC:15997]
ENSG000(	-1.3968	0.02794	-1.6733	0.00934	-1.54	LRBA	LPS-responsive vesicle trafficking, beach and anchor containing [Source:HGNC Symbol;Acc:HGNC:1742]
ENSG000(	-1.4268	0.01061	-1.635	0.00444	-1.53	ATP2B4	ATPase, Ca++ transporting, plasma membrane 4 [Source:HGNC Symbol;Acc:HGNC:817]
ENSG000(	-1.3485	0.02383	-1.7026	0.00552	-1.53	ZBTB38	zinc finger and BTB domain containing 38 [Source:HGNC Symbol;Acc:HGNC:26636]
ENSG000(	-1.4434	0.04151	-1.598	0.01786	-1.52	RBM47	RNA binding motif protein 47 [Source:HGNC Symbol;Acc:HGNC:30358]
ENSG000(	-1.5589	0.00021	-1.4703	0.00031	-1.51	CYP1B1	cytochrome P450, family 1, subfamily B, polypeptide 1 [Source:HGNC Symbol;Acc:HGNC:2597]
ENSG000(	-1.345	0.01801	-1.6421	0.00504	-1.49	AMOTL1	angiomotin like 1 [Source:HGNC Symbol;Acc:HGNC:17811]
ENSG000(	-1.5354	0.04441	-1.4509	0.04475	-1.49	ZNF292	zinc finger protein 292 [Source:HGNC Symbol;Acc:HGNC:18410]
ENSG000(	-1.315	0.04152	-1.6353	0.01136	-1.48	RIF1	replication timing regulatory factor 1 [Source:HGNC Symbol;Acc:HGNC:23207]
ENSG000(	-1.4234	0.0175	-1.5243	0.01019	-1.47	H6PD	hexose-6-phosphate dehydrogenase (glucose 1-dehydrogenase) [Source:HGNC Symbol;Acc:HGNC:4795]
ENSG000(	-1.6248	0.00802	-1.3222	0.01592	-1.47	PTK2B	protein tyrosine kinase 2 beta [Source:HGNC Symbol;Acc:HGNC:9612]
ENSG000(	-1.3259	0.02964	-1.6175	0.00935	-1.47	HUWE1	HECT, UBA and WWE domain containing 1, E3 ubiquitin protein ligase [Source:HGNC Symbol;Acc:HGNC:30892]
ENSG000(	-1.4781	0.03083	-1.456	0.02929	-1.47	RLIM	ring finger protein, LIM domain interacting [Source:HGNC Symbol;Acc:HGNC:13429]
ENSG000(	-1.384	0.0165	-1.5402	0.00839	-1.46	SPRED1	sprouty-related, EVH1 domain containing 1 [Source:HGNC Symbol;Acc:HGNC:20249]
ENSG000(	-1.3014	0.02402	-1.6142	0.0059	-1.46	ATF7IP	activating transcription factor 7 interacting protein [Source:HGNC Symbol;Acc:HGNC:20092]
ENSG000(	-1.482	0.02402	-1.4255	0.02558	-1.45	AKAP13	A kinase (PRKA) anchor protein 13 [Source:HGNC Symbol;Acc:HGNC:371]
ENSG000(	-1.2492	0.00703	-1.6568	0.00019	-1.45	KLF13	Kruppel-like factor 13 [Source:HGNC Symbol;Acc:HGNC:13672]
ENSG000(	-1.2828	0.03132	-1.6102	0.00792	-1.45	ABHD2	abhydrolase domain containing 2 [Source:HGNC Symbol;Acc:HGNC:18717]
ENSG000(	-1.2789	0.03756	-1.6139	0.00979	-1.45	DYNC1H1	dynein, cytoplasmic 1, heavy chain 1 [Source:HGNC Symbol;Acc:HGNC:2961]
ENSG000(	-1.1787	0.02642	-1.6775	0.00205	-1.43	ZMIZ1	zinc finger, MIZ-type containing 1 [Source:HGNC Symbol;Acc:HGNC:16493]
ENSG000(	-1.2936	0.02171	-1.5573	0.00718	-1.43	MKI67	marker of proliferation Ki-67 [Source:HGNC Symbol;Acc:HGNC:7107]
ENSG000(	-1.056	0.04689	-1.789	0.00087	-1.42	PVRL1	poliovirus receptor-related 1 (herpesvirus entry mediator C) [Source:HGNC Symbol;Acc:HGNC:9706]
ENSG000(	-1.2118	0.04473	-1.6021	0.00861	-1.41	WNK1	WNK lysine deficient protein kinase 1 [Source:HGNC Symbol;Acc:HGNC:14540]
ENSG000(	-1.2478	0.03207	-1.5573	0.00737	-1.40	APOOL	apolipoprotein O-like [Source:HGNC Symbol;Acc:HGNC:24009]
ENSG000(	-1.2195	0.03756	-1.5806	0.00798	-1.40	ARHGEF1	Rho guanine nucleotide exchange factor (GEF) 12 [Source:HGNC Symbol;Acc:HGNC:14193]
ENSG000(	-1.2147	0.0439	-1.5673	0.00949	-1.39	SETX	senataxin [Source:HGNC Symbol;Acc:HGNC:445]
ENSG000(	-1.259	0.0392	-1.5202	0.01148	-1.39	CASP2	caspase 2, apoptosis-related cysteine peptidase [Source:HGNC Symbol;Acc:HGNC:1503]
ENSG000(	-1.2778	0.03133	-1.49	0.01224	-1.38	ARHGAP5	Rho GTPase activating protein 5 [Source:HGNC Symbol;Acc:HGNC:675]
ENSG000(	-1.5948	0.01732	-1.1668	0.04819	-1.38	PRRG4	proline rich Gla (G-carboxyglutamic acid) 4 (transmembrane) [Source:HGNC Symbol;Acc:HGNC:30799]
ENSG000(	-1.5026	0.00157	-1.2532	0.00705	-1.38	TNS4	tensin 4 [Source:HGNC Symbol;Acc:HGNC:24352]
ENSG000(	-1.617	0.01023	-1.1306	0.04616	-1.37	CDH1	cadherin 1, type 1 [Source:HGNC Symbol;Acc:HGNC:1748]
ENSG000(	-1.289	0.02733	-1.4479	0.01255	-1.37	ELMSAN1	ELM2 and Myb/SANT-like domain containing 1 [Source:HGNC Symbol;Acc:HGNC:19853]
ENSG000(	-1.2273	0.02603	-1.5036	0.00737	-1.37	NFIC	nuclear factor I/C (CCAAT-binding transcription factor) [Source:HGNC Symbol;Acc:HGNC:7786]
ENSG000(	-1.1655	0.0392	-1.5627	0.00633	-1.36	BCL9L	B-cell CLL/lymphoma 9-like [Source:HGNC Symbol;Acc:HGNC:23688]
ENSG000(	-1.3252	0.00036	-1.3947	0.00011	-1.36	FLNA	filamin A, alpha [Source:HGNC Symbol;Acc:HGNC:3754]
ENSG000(	-1.1931	0.00925	-1.5225	0.00081	-1.36	TLE3	transducin-like enhancer of split 3 [Source:HGNC Symbol;Acc:HGNC:11839]
ENSG000(	-1.2135	0.01386	-1.4817	0.00305	-1.35	SOX9	SRY (sex determining region Y)-box 9 [Source:HGNC Symbol;Acc:HGNC:11204]
ENSG000(	-1.2459	0.02318	-1.4471	0.00918	-1.35	DLG5	discs, large homolog 5 (Drosophila) [Source:HGNC Symbol;Acc:HGNC:2904]
ENSG000(	-1.1462	0.01244	-1.5409	0.00101	-1.34	PLEC	plectin [Source:HGNC Symbol;Acc:HGNC:9069]
ENSG000(	-1.2323	0.00475	-1.4428	0.00081	-1.34	FLNB	filamin B, beta [Source:HGNC Symbol;Acc:HGNC:3755]
ENSG000(	-1.1825	0.01032	-1.4801	0.00152	-1.33	MICAL2	microtubule associated monooxygenase, calponin and LIM domain containing 2 [Source:HGNC Symbol;Acc:HGNC:24693]
ENSG000(	-1.2265	0.01072	-1.4349	0.00342	-1.33	ARHGAP3	Rho GTPase activating protein 35 [Source:HGNC Symbol;Acc:HGNC:4591]
ENSG000(	-1.1506	0.04319	-1.4889	0.00793	-1.32	HELZ2	helicase with zinc finger 2, transcriptional coactivator [Source:HGNC Symbol;Acc:HGNC:30021]
ENSG000(	-1.2132	0.02016	-1.4236	0.00772	-1.32	SLC7A1	solute carrier family 7 (cationic amino acid transporter, y+ system), member 1 [Source:HGNC Symbol;Acc:HGNC:11057]
ENSG000(	-1.2435	0.03845	-1.3883	0.01508	-1.32	AKR1C1	aldo-keto reductase family 1, member C1 [Source:HGNC Symbol;Acc:HGNC:384]
ENSG000(	-1.2233	0.02891	-1.3987	0.01266	-1.31	BBX	bobby sox homolog (Drosophila) [Source:HGNC Symbol;Acc:HGNC:14422]
ENSG000(	-1.1495	0.0278	-1.4685	0.00609	-1.31	CDCP1	CUB domain containing protein 1 [Source:HGNC Symbol;Acc:HGNC:24357]
ENSG000(	-1.3259	0.02603	-1.2904	0.01993	-1.31	ZNF703	zinc finger protein 703 [Source:HGNC Symbol;Acc:HGNC:25883]
ENSG000(	-1.1162	0.04234	-1.4769	0.00797	-1.30	LARP1	La ribonucleoprotein domain family, member 1 [Source:HGNC Symbol;Acc:HGNC:29531]
ENSG000(	-1.1622	0.0131	-1.4222	0.0027	-1.29	SYNJ2	synaptojanin 2 [Source:HGNC Symbol;Acc:HGNC:11504]
ENSG000(	-1.2827	0.0021	-1.277	0.00089	-1.28	ADGRG1	adhesion G protein-coupled receptor G1 [Source:HGNC Symbol;Acc:HGNC:4512]
ENSG000(	-1.1777	0.01428	-1.3691	0.00564	-1.27	SETD7	SET domain containing (lysine methyltransferase) 7 [Source:HGNC Symbol;Acc:HGNC:30412]
ENSG000(	-1.1478	0.01148	-1.3964	0.00289	-1.27	ITPR3	inositol 1,4,5-trisphosphate receptor, type 3 [Source:HGNC Symbol;Acc:HGNC:6182]
ENSG000(	-1.0269	0.04089	-1.505	0.00302	-1.27	PODXL	podocalyxin-like [Source:HGNC Symbol;Acc:HGNC:9171]
ENSG000(	-1.1747	0.00985	-1.3139	0.00473	-1.24	BAZ1B	bromodomain adjacent to zinc finger domain, 1B [Source:HGNC Symbol;Acc:HGNC:961]
ENSG000(	-1.0804	0.05	-1.4075	0.01026	-1.24	NSD1	nuclear receptor binding SET domain protein 1 [Source:HGNC Symbol;Acc:HGNC:14234]
ENSG000(	-1.1774	0.00514	-1.3051	0.0013	-1.24	ARRDC3	arrestin domain containing 3 [Source:HGNC Symbol;Acc:HGNC:29263]
ENSG000(	-1.0754	0.03322	-1.4047	0.00571	-1.24	USP38	ubiquitin specific peptidase 38 [Source:HGNC Symbol;Acc:HGNC:20067]
ENSG000(	-1.239	0.02379	-1.229	0.01823	-1.23	SPRY4	sprouty RTK signaling antagonist 4 [Source:HGNC Symbol;Acc:HGNC:15533]
ENSG000(	-1.1038	0.01032	-1.3623	0.00207	-1.23	TLN1	talin 1 [Source:HGNC Symbol;Acc:HGNC:11845]
ENSG000(	-1.1376	0.01359	-1.3265	0.00491	-1.23	SEMA3C	sema domain, immunoglobulin domain (Ig), short basic domain, secreted, (semaphorin) 3C [Source:HGNC Symbol;Acc:HGNC:10725]
ENSG000(	-1.0644	0.02146	-1.3929	0.00317	-1.23	SLK	STE20-like kinase [Source:HGNC Symbol;Acc:HGNC:11088]
ENSG000(	-1.1033	0.04323	-1.3349	0.01356	-1.22	CNOT1	CCR4-NOT transcription complex, subunit 1 [Source:HGNC Symbol;Acc:HGNC:7877]
ENSG000(	-1.2034	0.0323	-1.2227	0.02227	-1.21	AMMECR1	Alport syndrome, mental retardation, midface hypoplasia and elliptocytosis chromosomal region gene 1 [Source:HGNC Symbol;Acc:HGNC:467]
ENSG000(	-1.1204	0.04662	-1.2859	0.01837	-1.20	TCF20	transcription factor 20 (AR1) [Source:HGNC Symbol;Acc:HGNC:11631]
ENSG000(	-1.0513	0.02733	-1.3442	0.00565	-1.20	SH3PXD2A	SH3 and PX domains 2A [Source:HGNC Symbol;Acc:HGNC:23664]
ENSG000(	-0.9864	0.04003	-1.3991	0.004	-1.19	IGF2R	insulin-like growth factor 2 receptor [Source:HGNC Symbol;Acc:HGNC:5467]
ENSG000(	-1.0193	0.0471	-1.3593	0.00772	-1.19	TRERF1	transcriptional regulating factor 1 [Source:HGNC Symbol;Acc:HGNC:18273]
ENSG000(	-0.9691	0.01803	-1.4029	0.00059	-1.19	STC1	stanniocalcin 1 [Source:HGNC Symbol;Acc:HGNC:11373]
ENSG000(	-1.083	0.02	-1.2748	0.00552	-1.18	ARHGEF5	Rho guanine nucleotide exchange factor (GEF) 5 [Source:HGNC Symbol;Acc:HGNC:13209]
ENSG000(	-1.1262	0.00664	-1.1998	0.00351	-1.16	ACSL4	acyl-CoA synthetase long-chain family member 4 [Source:HGNC Symbol;Acc:HGNC:3571]
ENSG000(	-1.028	0.00684	-1.2941	0.00065	-1.16	MYH9	myosin, heavy chain 9, non-muscle [Source:HGNC Symbol;Acc:HGNC:7579]
ENSG000(	-1.1828	0.00915	-1.1384	0.01019	-1.16	FAM199X	family with sequence similarity 199, X-linked [Source:HGNC Symbol;Acc:HGNC:25195]
ENSG000(	-1.0517	0.04845	-1.2612	0.01497	-1.16	MECP2	methyl CpG binding protein 2 [Source:HGNC Symbol;Acc:HGNC:6990]
ENSG000(	-0.9041	0.02545	-1.405	0.00049	-1.15	SGK223	Tyrosine-protein kinase SgK223 [Source:UniProtKB/Swiss-Prot;Acc:Q86YV5]
ENSG000(	-1.0626	0.04234	-1.2428	0.0147	-1.15	FAM84B	family with sequence similarity 84, member B [Source:HGNC Symbol;Acc:HGNC:24166]
ENSG000(	-1.1132	0.02574	-1.1918	0.01658	-1.15	PRKAR2A	protein kinase, cAMP-dependent, regulatory, type II, alpha [Source:HGNC Symbol;Acc:HGNC:9391]
ENSG000(	-1.0078	0.02402	-1.2951	0.00473	-1.15	KIAA0100	KIAA0100 [Source:HGNC Symbol;Acc:HGNC:28960]
ENSG000(	-1.1129	0.01739	-1.1865	0.01132	-1.15	THOC2	THO complex 2 [Source:HGNC Symbol;Acc:HGNC:19073]
ENSG000(	-1.0607	0.04533	-1.2254	0.01601	-1.14	ZBTB33	zinc finger and BTB domain containing 33 [Source:HGNC Symbol;Acc:HGNC:16682]
ENSG000(	-0.9507	0.02604	-1.3242	0.00223	-1.14	TNRC18	trinucleotide repeat containing 18 [Source:HGNC Symbol;Acc:HGNC:11962]
ENSG000(	-1.0166	0.03046	-1.2565	0.00863	-1.14	IQGAP1	IQ motif containing GTPase activating protein 1 [Source:HGNC Symbol;Acc:HGNC:6110]
ENSG000(	-0.9263	0.0429	-1.3259	0.00393	-1.13	ANKRD11	ankyrin repeat domain 11 [Source:HGNC Symbol;Acc:HGNC:21316]
ENSG000(	-0.9972	0.03171	-1.2547	0.00644	-1.13	TMEM164	transmembrane protein 164 [Source:HGNC Symbol;Acc:HGNC:26217]
ENSG000(	-0.9877	0.02679	-1.2593	0.00545	-1.12	STAG2	stromal antigen 2 [Source:HGNC Symbol;Acc:HGNC:11355]
ENSG000(	-0.9528	0.03572	-1.2923	0.00473	-1.12	CEP250	centrosomal protein 250kDa [Source:HGNC Symbol;Acc:HGNC:1859]
ENSG000(	-0.9752	0.01575	-1.2514	0.00252	-1.11	OGT	O-linked N-acetylglucosamine (GlcNAc) transferase [Source:HGNC Symbol;Acc:HGNC:8127]
ENSG000(	-1.0654	0.02568	-1.1595	0.01351	-1.11	KCTD12	potassium channel tetramerization domain containing 12 [Source:HGNC Symbol;Acc:HGNC:14678]
ENSG000(	-0.9682	0.03513	-1.2564	0.00644	-1.11	SKI	SKI proto-oncogene [Source:HGNC Symbol;Acc:HGNC:10896]
ENSG000(	-1.0229	0.00211	-1.1995	0.00011	-1.11	ZNF185	zinc finger protein 185 (LIM domain) [Source:HGNC Symbol;Acc:HGNC:12976]
ENSG000(	-1.0077	0.04424	-1.213	0.01197	-1.11	SLC9A6	solute carrier family 9, subfamily A (NHE6, cation proton antiporter 6), member 6 [Source:HGNC Symbol;Acc:HGNC:11079]
ENSG000(	-1.1127	0.04663	-1.1013	0.03405	-1.11	RSBN1L	round spermatid basic protein 1-like [Source:HGNC Symbol;Acc:HGNC:24765]
ENSG000(	-0.9391	0.01588	-1.2738	0.00103	-1.11	ST3GAL1	ST3 beta-galactoside alpha-2,3-sialyltransferase 1 [Source:HGNC Symbol;Acc:HGNC:10862]
ENSG000(	-1.0201	0.02773	-1.1918	0.00904	-1.11	MEGF8	multiple EGF-like-domains 8 [Source:HGNC Symbol;Acc:HGNC:3233]
ENSG000(	-1.0819	0.04845	-1.1285	0.0281	-1.11	CDK14	cyclin-dependent kinase 14 [Source:HGNC Symbol;Acc:HGNC:8883]
ENSG000(	-0.9258	0.01725	-1.2813	0.00108	-1.10	NUP205	nucleoporin 205kDa [Source:HGNC Symbol;Acc:HGNC:18658]
ENSG000(	-0.9437	0.00915	-1.2632	0.00038	-1.10	EFNB2	ephrin-B2 [Source:HGNC Symbol;Acc:HGNC:3227]
ENSG000(	-0.9888	0.02733	-1.2044	0.00702	-1.10	POLA1	polymerase (DNA directed), alpha 1, catalytic subunit [Source:HGNC Symbol;Acc:HGNC:9173]
ENSG000(	-0.9907	0.03756	-1.2004	0.01093	-1.10	ZMYM4	zinc finger, MYM-type 4 [Source:HGNC Symbol;Acc:HGNC:13055]
ENSG000(	-0.9237	0.0457	-1.2652	0.0059	-1.09	TRIM56	tripartite motif containing 56 [Source:HGNC Symbol;Acc:HGNC:19028]
ENSG000(	-0.9002	0.03562	-1.2832	0.00305	-1.09	C6orf62	chromosome 6 open reading frame 62 [Source:HGNC Symbol;Acc:HGNC:20998]
ENSG000(	-0.9232	0.04983	-1.252	0.00772	-1.09	RBPJ	recombination signal binding protein for immunoglobulin kappa J region [Source:HGNC Symbol;Acc:HGNC:5724]
ENSG000(	-1.0984	0.01478	-1.0741	0.01293	-1.09	MUC1	mucin 1, cell surface associated [Source:HGNC Symbol;Acc:HGNC:7508]
ENSG000(	-0.9087	0.03121	-1.2524	0.0032	-1.08	NDST1	N-deacetylase/N-sulfotransferase (heparan glucosaminyl) 1 [Source:HGNC Symbol;Acc:HGNC:7680]
ENSG000(	-0.9538	0.01023	-1.2051	0.0013	-1.08	PTPRF	protein tyrosine phosphatase, receptor type, F [Source:HGNC Symbol;Acc:HGNC:9670]
ENSG000(	-0.954	0.04392	-1.2	0.00924	-1.08	MAMLD1	mastermind-like domain containing 1 [Source:HGNC Symbol;Acc:HGNC:2568]
ENSG000(	-1.1309	0.02016	-1.0159	0.03053	-1.07	PIK3R1	phosphoinositide-3-kinase, regulatory subunit 1 (alpha) [Source:HGNC Symbol;Acc:HGNC:8979]
ENSG000(	-0.9842	0.03714	-1.1569	0.01148	-1.07	DSE	dermatan sulfate epimerase [Source:HGNC Symbol;Acc:HGNC:21144]
ENSG000(	-0.8404	0.03901	-1.2967	0.00132	-1.07	RPTOR	regulatory associated protein of MTOR, complex 1 [Source:HGNC Symbol;Acc:HGNC:30287]
ENSG000(	-0.9387	0.02244	-1.189	0.00473	-1.06	ADAR	adenosine deaminase, RNA-specific [Source:HGNC Symbol;Acc:HGNC:225]
ENSG000(	-0.9375	0.04307	-1.1899	0.01026	-1.06	ARHGAP2	Rho GTPase activating protein 29 [Source:HGNC Symbol;Acc:HGNC:30207]
ENSG000(	-0.9814	0.02104	-1.141	0.00713	-1.06	MTMR1	myotubularin related protein 1 [Source:HGNC Symbol;Acc:HGNC:7449]
ENSG000(	-0.9665	0.0366	-1.153	0.0111	-1.06	ZBTB4	zinc finger and BTB domain containing 4 [Source:HGNC Symbol;Acc:HGNC:23847]
ENSG000(	-0.8366	0.03735	-1.2697	0.00163	-1.05	ZFR	zinc finger RNA binding protein [Source:HGNC Symbol;Acc:HGNC:17277]
ENSG000(	-1.0401	0.03699	-1.0656	0.02572	-1.05	MOCOS	molybdenum cofactor sulfurase [Source:HGNC Symbol;Acc:HGNC:18234]
ENSG000(	-0.9575	0.01436	-1.1449	0.00351	-1.05	MYO18A	myosin XVIIIA [Source:HGNC Symbol;Acc:HGNC:31104]
ENSG000(	-0.8084	0.00617	-1.2739	8.8E-07	-1.04	ITGA5	integrin, alpha 5 (fibronectin receptor, alpha polypeptide) [Source:HGNC Symbol;Acc:HGNC:6141]
ENSG000(	-0.9385	0.01507	-1.136	0.00351	-1.04	POM121C	POM121 transmembrane nucleoporin C [Source:HGNC Symbol;Acc:HGNC:34005]
ENSG000(	-1.1423	0.01226	-0.9316	0.02821	-1.04	L1CAM	L1 cell adhesion molecule [Source:HGNC Symbol;Acc:HGNC:6470]
ENSG000(	-0.7907	0.04927	-1.2733	0.00136	-1.03	SSFA2	sperm specific antigen 2 [Source:HGNC Symbol;Acc:HGNC:11319]
ENSG000(	-0.9212	0.01094	-1.133	0.00211	-1.03	PLXNA1	plexin A1 [Source:HGNC Symbol;Acc:HGNC:9099]
ENSG000(	-0.9686	0.01394	-1.0792	0.00544	-1.02	SLC37A2	solute carrier family 37 (glucose-6-phosphate transporter), member 2 [Source:HGNC Symbol;Acc:HGNC:20644]
ENSG000(	-0.8689	0.02547	-1.1662	0.00289	-1.02	PLXNA3	plexin A3 [Source:HGNC Symbol;Acc:HGNC:9101]
ENSG000(	-0.8558	0.04613	-1.1726	0.00648	-1.01	PRRC2A	proline-rich coiled-coil 2A [Source:HGNC Symbol;Acc:HGNC:13918]
ENSG000(	-0.9225	0.0215	-1.1045	0.00588	-1.01	TGFA	transforming growth factor, alpha [Source:HGNC Symbol;Acc:HGNC:11765]
ENSG000(	-0.9314	0.03019	-1.0765	0.01131	-1.00	YES1	YES proto-oncogene 1, Src family tyrosine kinase [Source:HGNC Symbol;Acc:HGNC:12841]
ENSG000(	-0.9464	0.03198	-1.0591	0.01354	-1.00	TCAF1	TRPM8 channel-associated factor 1 [Source:HGNC Symbol;Acc:HGNC:22201]
ENSG000(	-0.8898	0.03714	-1.1044	0.00827	-1.00	MFSD6	major facilitator superfamily domain containing 6 [Source:HGNC Symbol;Acc:HGNC:24711]
ENSG000(	-0.7873	0.00912	-1.1974	1.8E-05	-0.99	ESYT2	extended synaptotagmin-like protein 2 [Source:HGNC Symbol;Acc:HGNC:22211]
ENSG000(	-0.7976	0.02003	-1.1836	0.00049	-0.99	INTS1	integrator complex subunit 1 [Source:HGNC Symbol;Acc:HGNC:24555]
ENSG000(	-0.7974	0.00763	-1.1828	1.7E-05	-0.99	ITGB4	integrin, beta 4 [Source:HGNC Symbol;Acc:HGNC:6158]
ENSG000(	-0.9057	0.03248	-1.0693	0.01132	-0.99	RAD50	RAD50 homolog, double strand break repair protein [Source:HGNC Symbol;Acc:HGNC:9816]
ENSG000(	-0.8463	0.03916	-1.1141	0.0063	-0.98	CHD2	chromodomain helicase DNA binding protein 2 [Source:HGNC Symbol;Acc:HGNC:1917]
ENSG000(	-0.8729	0.03719	-1.064	0.00924	-0.97	ELF4	E74-like factor 4 (ets domain transcription factor) [Source:HGNC Symbol;Acc:HGNC:3319]
ENSG000(	-0.7774	0.02547	-1.1583	0.00095	-0.97	SLC38A2	solute carrier family 38, member 2 [Source:HGNC Symbol;Acc:HGNC:13448]
ENSG000(	-0.9054	0.00988	-1.0184	0.0033	-0.96	EPAS1	endothelial PAS domain protein 1 [Source:HGNC Symbol;Acc:HGNC:3374]
ENSG000(	-0.8488	0.04931	-1.0736	0.01019	-0.96	SOCS6	suppressor of cytokine signaling 6 [Source:HGNC Symbol;Acc:HGNC:16833]
ENSG000(	-0.8863	0.00022	-1.0013	4.8E-06	-0.94	VEGFA	vascular endothelial growth factor A [Source:HGNC Symbol;Acc:HGNC:12680]
ENSG000(	-0.8511	0.01787	-1.0348	0.00482	-0.94	CHD4	chromodomain helicase DNA binding protein 4 [Source:HGNC Symbol;Acc:HGNC:1919]
ENSG000(	-0.7912	0.04717	-1.0868	0.00613	-0.94	PPP3CA	protein phosphatase 3, catalytic subunit, alpha isozyme [Source:HGNC Symbol;Acc:HGNC:9314]
ENSG000(	-0.9754	0.01749	-0.897	0.02114	-0.94	RAP2C	RAP2C, member of RAS oncogene family [Source:HGNC Symbol;Acc:HGNC:21165]
ENSG000(	-0.7702	0.02813	-1.0929	0.00219	-0.93	YWHAG	tyrosine 3-monooxygenase/tryptophan 5-monooxygenase activation protein, gamma [Source:HGNC Symbol;Acc:HGNC:12852]
ENSG000(	-0.8787	0.0323	-0.9659	0.01395	-0.92	PHKA1	phosphorylase kinase, alpha 1 (muscle) [Source:HGNC Symbol;Acc:HGNC:8925]
ENSG000(	-0.828	0.00604	-1.0135	0.00051	-0.92	TGM2	transglutaminase 2 [Source:HGNC Symbol;Acc:HGNC:11778]
ENSG000(	-0.8355	0.01761	-0.9981	0.00483	-0.92	TNPO3	transportin 3 [Source:HGNC Symbol;Acc:HGNC:17103]
ENSG000(	-0.7325	0.04848	-1.0998	0.00288	-0.92	SNX19	sorting nexin 19 [Source:HGNC Symbol;Acc:HGNC:21532]
ENSG000(	-0.7684	0.02733	-1.03	0.00315	-0.90	FOSL2	FOS-like antigen 2 [Source:HGNC Symbol;Acc:HGNC:3798]
ENSG000(	-0.7784	0.01927	-1.0087	0.00223	-0.89	PC	pyruvate carboxylase [Source:HGNC Symbol;Acc:HGNC:8636]
ENSG000(	-0.8273	0.0169	-0.9597	0.00636	-0.89	MTPN	myotrophin [Source:HGNC Symbol;Acc:HGNC:15667]
ENSG000(	-0.8048	0.04009	-0.9786	0.01001	-0.89	CHST15	carbohydrate (N-acetylgalactosamine 4-sulfate 6-O) sulfotransferase 15 [Source:HGNC Symbol;Acc:HGNC:18137]
ENSG000(	-0.7173	0.04233	-1.0491	0.00275	-0.88	MAGT1	magnesium transporter 1 [Source:HGNC Symbol;Acc:HGNC:28880]
ENSG000(	-0.7838	0.04409	-0.9653	0.01148	-0.87	JADE2	jade family PHD finger 2 [Source:HGNC Symbol;Acc:HGNC:22984]
ENSG000(	-0.8057	0.01118	-0.9378	0.00294	-0.87	FAM83A	family with sequence similarity 83, member A [Source:HGNC Symbol;Acc:HGNC:28210]
ENSG000(	-0.7368	0.04983	-1.001	0.00734	-0.87	NAA15	N(alpha)-acetyltransferase 15, NatA auxiliary subunit [Source:HGNC Symbol;Acc:HGNC:30782]
ENSG000(	-0.5529	0.04562	-1.1845	1.2E-06	-0.87	NDRG1	N-myc downstream regulated 1 [Source:HGNC Symbol;Acc:HGNC:7679]
ENSG000(	-0.7497	0.0323	-0.9652	0.00636	-0.86	UBE3C	ubiquitin protein ligase E3C [Source:HGNC Symbol;Acc:HGNC:16803]
ENSG000(	-0.5846	0.0499	-1.1132	6.8E-05	-0.85	RRBP1	ribosome binding protein 1 [Source:HGNC Symbol;Acc:HGNC:10448]
ENSG000(	-0.7223	0.03192	-0.9735	0.00342	-0.85	FURIN	furin (paired basic amino acid cleaving enzyme) [Source:HGNC Symbol;Acc:HGNC:8568]
ENSG000(	-0.6513	0.0411	-1.0314	0.00111	-0.84	SCD	stearoyl-CoA desaturase (delta-9-desaturase) [Source:HGNC Symbol;Acc:HGNC:10571]
ENSG000(	-0.7022	0.03263	-0.9792	0.00302	-0.84	TRIM25	tripartite motif containing 25 [Source:HGNC Symbol;Acc:HGNC:12932]
ENSG000(	-0.6686	0.04773	-0.9789	0.00369	-0.82	SLC7A5	solute carrier family 7 (amino acid transporter light chain, L system), member 5 [Source:HGNC Symbol;Acc:HGNC:11063]
ENSG000(	-0.5877	0.04773	-1.0081	0.00049	-0.80	B4GALT1	UDP-Gal:betaGlcNAc beta 1,4- galactosyltransferase, polypeptide 1 [Source:HGNC Symbol;Acc:HGNC:924]
ENSG000(	-0.6808	0.02499	-0.9129	0.00294	-0.80	GTF2IP1	general transcription factor IIi pseudogene 1 [Source:HGNC Symbol;Acc:HGNC:4660]
ENSG000(	-0.6653	0.03164	-0.9096	0.00305	-0.79	EPHB4	EPH receptor B4 [Source:HGNC Symbol;Acc:HGNC:3395]
ENSG000(	-0.6227	0.02092	-0.8915	0.00081	-0.76	ABLIM3	actin binding LIM protein family, member 3 [Source:HGNC Symbol;Acc:HGNC:29132]
ENSG000(	-0.8312	0.00862	-0.6288	0.0343	-0.73	PMEPA1	prostate transmembrane protein, androgen induced 1 [Source:HGNC Symbol;Acc:HGNC:14107]
ENSG000(	-0.5073	0.04473	-0.8225	0.00095	-0.66	ITGA3	integrin, alpha 3 (antigen CD49C, alpha 3 subunit of VLA-3 receptor) [Source:HGNC Symbol;Acc:HGNC:6139]
ENSG000(	-0.5595	0.02529	-0.7382	0.00319	-0.65	IRAK1	interleukin-1 receptor-associated kinase 1 [Source:HGNC Symbol;Acc:HGNC:6112]
