# Supplementary material for: Anillin regulates breast cancer cell migration, growth, and metastasis by non-canonical mechanisms involving control of cell stemness and differentiation
Source: Breast Cancer Res. 2020 Jan 7;22:3. doi: 10.1186/s13058-019-1241-x (PMC6947866; doi:10.1186/s13058-019-1241-x)
Supplement: Supplementary file 14 — Figure S11. Loss of anillin causes transcriptional reprogramming of breast cancer cells. RNA sequencing analysis was performed on mRNA samples isolated from control and anillin-depleted (sgRNA 3 and sgRNA 4) MDA-MB-231 cells. Gene Set enrichment analysis was performed on 275 differentially expressed genes common for two CRISPR sgRNA-derived cell lines. Red and blue bars depict cellular processes upregulated and downregulated in anillin-deficient cells, respectively. [file 13058_2019_1241_MOESM14_ESM.pptx]

## Slide 1
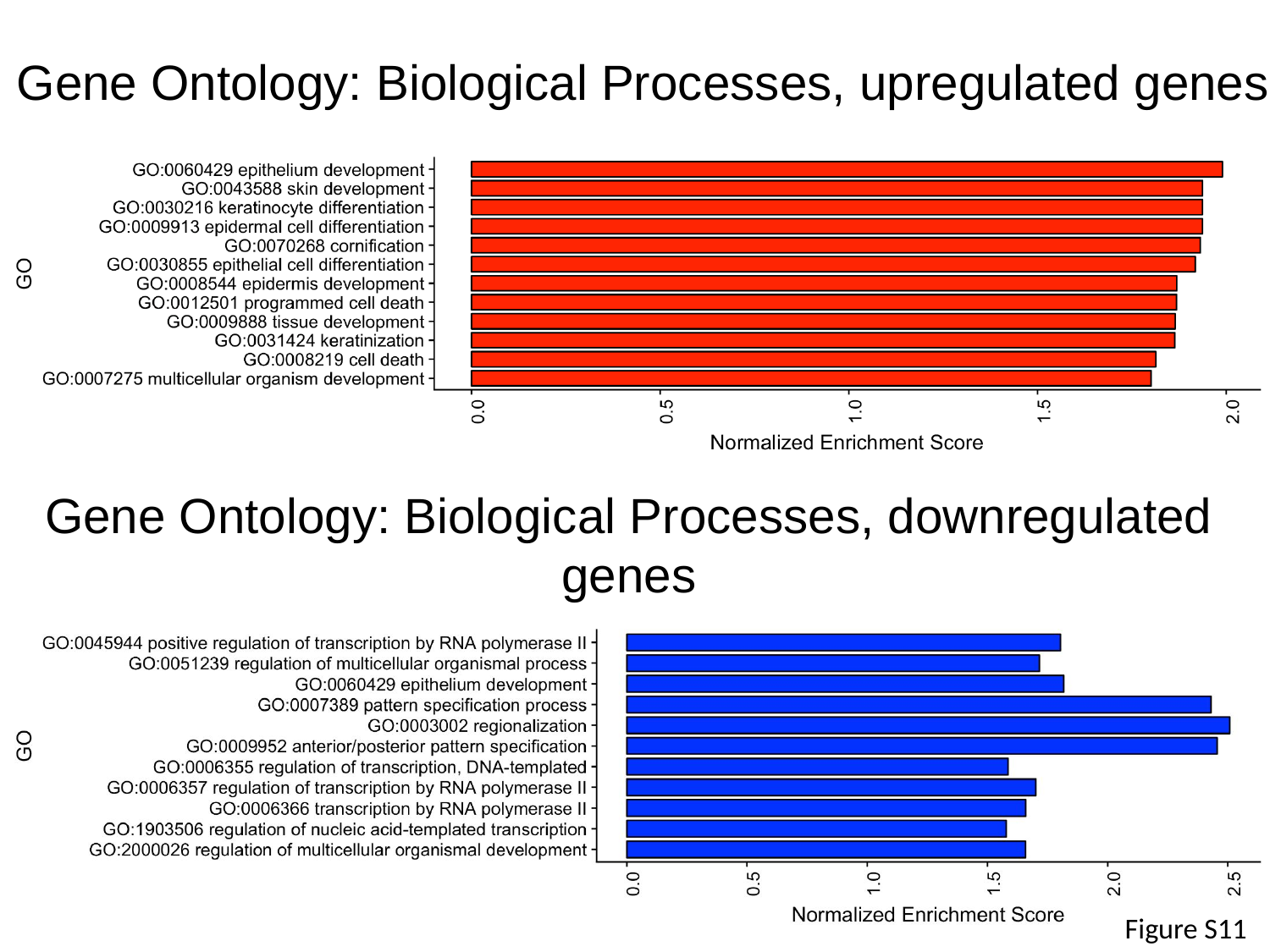

Gene Ontology: Biological Processes, upregulated genes
Gene Ontology: Biological Processes, downregulated genes
Figure S11
