# Supplementary material for: Anillin regulates breast cancer cell migration, growth, and metastasis by non-canonical mechanisms involving control of cell stemness and differentiation
Source: Breast Cancer Res. 2020 Jan 7;22:3. doi: 10.1186/s13058-019-1241-x (PMC6947866; doi:10.1186/s13058-019-1241-x)
Supplement: Supplementary file 15 — Figure S12. Downregulation of P-cadherin expression does not reverse the decreased motility of anillin-deficient breast cancer cells. P-cadherin was transiently depleted in control and anillin-deficient MDA-MB-231 cells by a selective siRNA SmartPool. (A) Immunoblotting analysis shows the efficiency of P-cadherin depletion. (B,C) Representative images and quantification of wound healing in control and anillin-deficient cell monolayers with and without P-cadherin depletion. Scale bar, 100 μm. (D,E) Representative images and quantification analysis of Matrigel invasion of control and anillin-overexpressing MDA-MB-231 cells transfected with either control or P-cadherin-specific siRNAs. Data are presented as mean ± SE (n = 3). Scale bar, 50 μm. [file 13058_2019_1241_MOESM15_ESM.pptx]

## Slide 1
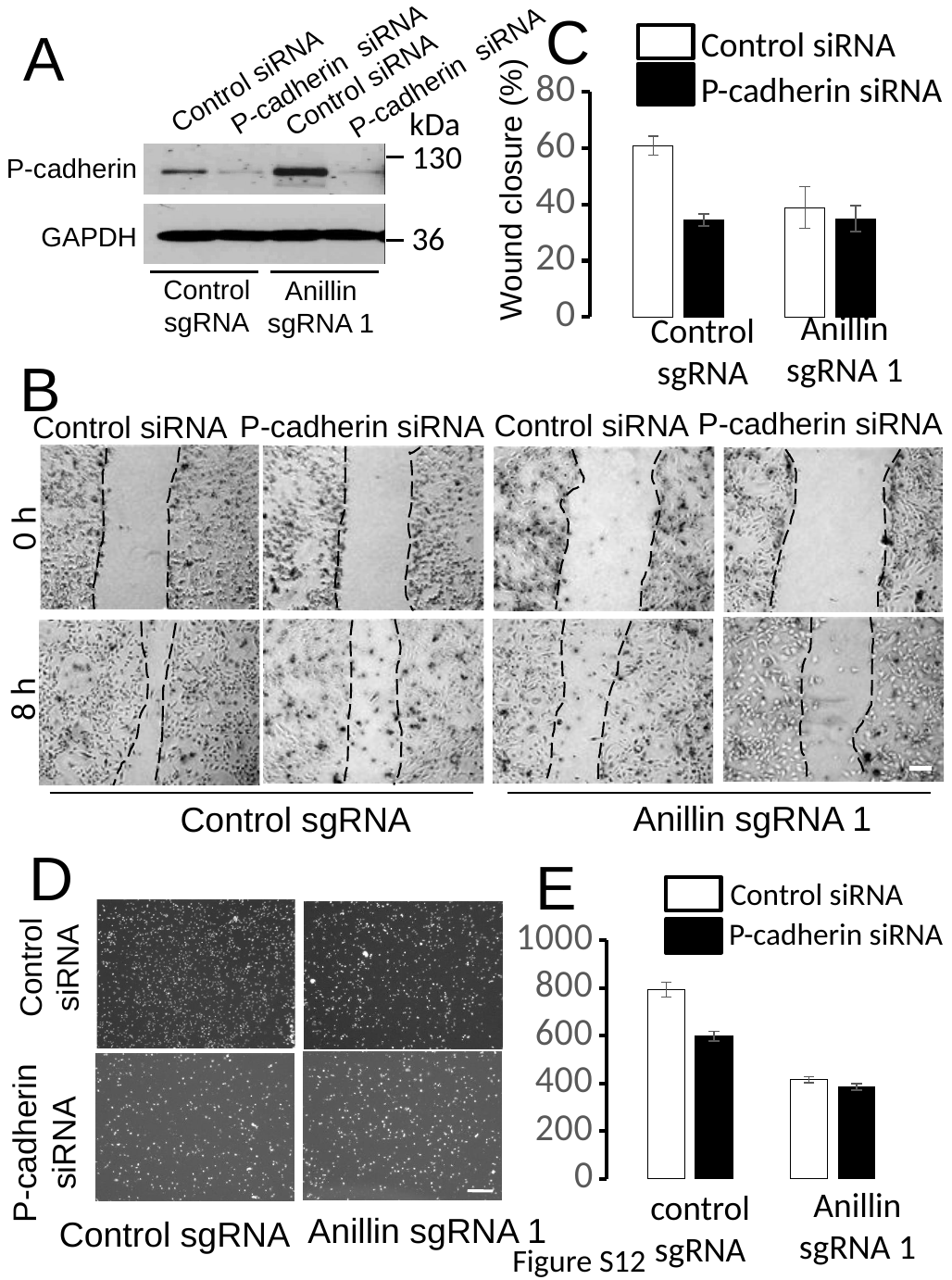

C
A
Control siRNA
P-cadherin siRNA
P-cadherin siRNA
Control siRNA
Control siRNA
P-cadherin siRNA
### Chart
| Category | | | | | |
|---|---|---|---|---|---|
kDa
130
P-cadherin
Wound closure (%)
36
GAPDH
Control sgRNA
Anillin sgRNA 1
Anillin sgRNA 1
Control sgRNA
B
P-cadherin siRNA
Control siRNA
P-cadherin siRNA
Control siRNA
0 h
8 h
Anillin sgRNA 1
Control sgRNA
D
E
Control siRNA
P-cadherin siRNA
### Chart
| Category | | | | | |
|---|---|---|---|---|---|
Control siRNA
P-cadherin siRNA
Anillin sgRNA 1
control sgRNA
Anillin sgRNA 1
Control sgRNA
Figure S12
